# Characterising side chains in large proteins by protonless ^13^C-detected NMR spectroscopy

**DOI:** 10.1038/s41467-019-09743-4

**Published:** 2019-04-15

**Authors:** Ruth B. Pritchard, D. Flemming Hansen

**Affiliations:** Institute of Structural and Molecular Biology, Division of Biosciences, University College London, London, UK WC1E 6BT

## Abstract

Side chains cover protein surfaces and are fundamental to processes as diverse as substrate recognition, protein folding and enzyme catalysis. However, characterisation of side-chain motions has so far been restricted to small proteins and methyl-bearing side chains. Here we present a class of methods, based on ^13^C-detected NMR spectroscopy, to more generally quantify motions and interactions of side chains in medium-to-large proteins. A single, uniformly isotopically labelled sample is sufficient to characterise the side chains of six different amino acid types. Side-chain conformational dynamics on the millisecond time-scale can be quantified by incorporating chemical exchange saturation transfer (CEST) into the presented methods, whilst long-range ^13^C-^13^C scalar couplings reporting on nanosecond to millisecond motions can be quantified in proteins as large as 80 kDa. The presented class of methods promises characterisation of side-chain behaviour at a level that has so far been reserved for the protein backbone.

## Abstract (web summary)

Analysis of side-chain motions by NMR has so far been restricted to small proteins and methyl-bearing side chains. Here, the authors present NMR methods based on ^13^C direct detection of highly deuterated protein samples that yield sharp and well-resolved signals and allow the characterisation of side-chain conformational dynamics of six different amino acid types in medium-to-large proteins.

## Introduction

Proteins are dynamic entities whose molecular function is intrinsically related to their structure and dynamic sampling, both in the immediate vicinity of active sites and in regulatory sites^1,2^. Proteins are often viewed as representations of their backbone and most experimental studies of protein dynamics to date have primarily focussed on the protein backbone, with much less attention paid to side chains^3–9^. Whilst knowledge of the behaviour of the protein backbone is essential in order to understand many aspects of protein function, bringing side chains into focus is crucial. It is the side chains that give the amino acids in proteins their unique chemical diversity, for example, side chains form critical parts of many active sites of enzymes and the side chains presented on the protein surface are key to substrate recognition and binding events. In many cases, the dynamics and interactions of side chains are as, if not more, important to the biological function than the overall backbone conformation. In addition, the motions of side chains are often decorrelated from the backbone^10–12^. In order to understand the mechanisms of enzymes and characterise macromolecular interactions and regulation, it is imperative to be able to specifically characterise the side-chain structure and movements.

Nuclear magnetic resonance (NMR) spectroscopy is uniquely situated to generally characterise the interactions and conformational sampling of side chains in proteins. However, most experimental studies of protein motions using NMR spectroscopy to date have focussed on the backbone or methyl groups, and detailed information regarding side-chain behaviour has been restricted to small proteins^13,14^ and methyl-bearing side chains^15–17^. The limitations of currently available approaches are mainly due to line broadening and signal loss as a result of efficient relaxation pathways, as well as insufficient resolution and signal overlap in the NMR correlation maps used to extract parameters reporting on dynamics and interactions.

Here, we present a class of NMR methods that allows a more general characterisation of side chains in medium-to-large proteins. The presented class of methods is anchored in ^13^C-direct detection^18^ NMR spectroscopy of per-deuterated proteins. The slow relaxation of the aliphatic ^13^C nuclei in deuterated proteins results in sharp NMR signals, whilst the correlation of two ^13^C chemical shifts results in high-resolution and well-resolved two-dimensional NMR spectra. It is shown that the slow transverse ^13^C relaxation rates enable a large range of NMR experiments to be performed to characterise the structure, interactions and dynamics of side chains in medium-to-large (< 82 kDa) proteins. The presented experiments include a quantification of long-range ^13^C–^13^C scalar couplings^13^ reporting on the sampling of side-chain dihedral angles and chemical exchange saturation transfer (CEST) experiments^19^ to characterise low-populated states.

## Results and discussion

### Side-chain ^13^C–^13^C correlation spectra

**Fig. 1:**
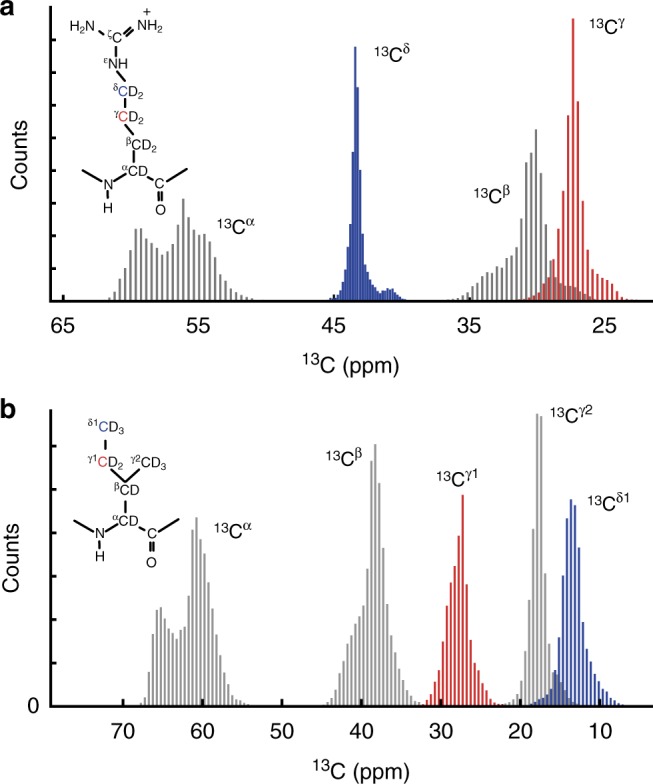
Examples of aliphatic ^13^C chemical shift distributions. Distribution of the assigned ^13^C chemical shifts of (**a**) arginine and isoleucine (**b**) residues extracted from the BMRB^20^ databank. Terminal ^13^C_t_, directly coupled to just one other ^13^C are coloured blue and the bonded, penultimate ^13^C_p_, are  coloured red. For clarity, only one of the two ^13^C_t_–^13^C_p_ pairs have been highlighted in isoleucine (See Supplementary Fig. 1 for other sites)

The BMRB^20^ databank provides a large database of assigned NMR chemical shifts, including the side-chain ^13^C chemical shift. Analysis of these data reveals six side chains (Fig. 1; Supplementary Fig. 1) with a ‘terminal’ ^13^C (^13^C_t_), which is directly bonded to just one other ^13^C and which has a chemical shift distribution that is isolated from its directly bonded penultimate ^13^C (^13^C_p_). This distinct chemical shift profile means that the terminal ^13^C_t_ can be selectively excited in an NMR experiment using frequency-selective pulses. Selective excitation enables spectral filtering and observation of specific residue types in multidimensional NMR spectra, even when using uniformly isotopically labelled proteins.

**Fig. 2:**
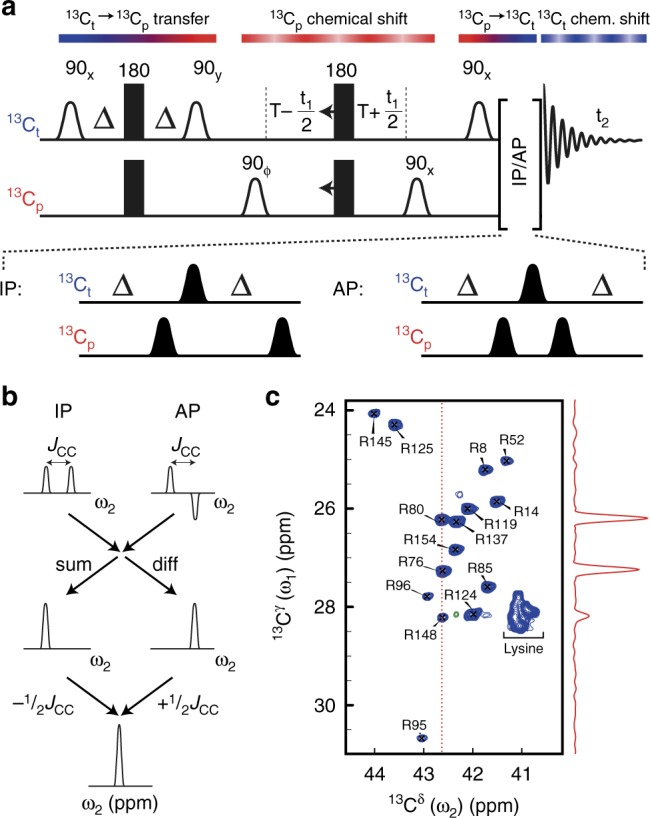
^13^C–^13^C side-chain correlation spectra of per-deuterated proteins. **a** Schematic representation of the NMR pulse sequence used to obtain ^13^C–^13^C side-chain correlation spectra. The flow of the magnetisation between ^13^C_t_ (blue) and ^13^C_p_ (red) is shown above the sequence with colour gradients. The following delays are used: Δ = 1/(4*J*_CC_) ≈ 7.1 ms, T = 1/(2*J*_CC_) ≈ 14.1 ms, where *J*_CC_ is the one-bond ^13^C–^13^C scalar coupling constant. Rectangular pulses are high-power and not selective, bell-shaped pulses are frequency selective (90°: white outlined, 180°: black). Deuterium, ^2^H, is decoupled throughout the sequence and frequency discrimination is obtained by states–TPPI of phase ϕ^21^. **b** Schematic representation of post-processing to obtain the decoupled spectrum. **c** Arginine ^13^C^δ^–^13^C^γ^ correlation of the 18-kDa protein T4L L99A, obtained on a 1.4 mM sample at a static field of 14.1 T at 278 K in 37 min

The core element of the presented side-chain-specific NMR experiments, Fig. 2a, correlates the terminal carbon ^13^C_t_ with its directly bonded penultimate ^13^C_p_ within a protein side chain. The terminal ^13^C_t_ includes ^13^C^δ^ in the arginine side chains, ^13^C^δ1^ and ^13^C^γ2^ in isoleucine, ^13^C^ε^ in lysine, ^13^C^δ^ in proline, ^13^C^γ2^ in threonine and ^13^C^γ^ in valine residues. The fact that the magnetisation of interest starts and is detected on the terminal ^13^C_t_ has some key advantages. The homonuclear coupling pattern for ^13^C_t_ is a simple doublet, which allows for the magnetisation of interest to be transferred completely between ^13^C_t_ and ^13^C_p_ using simple INEPTs and also facilitates virtual decoupling in the ^13^C_t_ detected dimension.

In the core experiment, Fig. 2a and Supplementary Fig. 2, magnetisation is initially transferred from ^13^C_t_ to ^13^C_p_, then labelled with the chemical shift of ^13^C_p_ during *t*_1_ and finally transferred back to ^13^C_t_ for detection. The doublet peak splitting observed for ^13^C_t_ because of the evolution of the single one-bond ^13^C_t_–^13^C_p_ scalar coupling during acquisition (*t*_2_) can conveniently be resolved by recording two sub-spectra, in-phase (IP) and anti-phase (AP)^22^. Figure 2b shows how subsequent post-processing is used to virtually ‘decouple’ the spectra in the direct ^13^C_t_ dimension, so that single peaks are observed in multidimensional correlation spectra. One-bond ^13^C–^13^C couplings in indirect dimensions (*t*_1_) are removed using constant-time evolutions^23^, and evolutions of the ^2^H–^13^C couplings are efficiently suppressed using ^2^H decoupling (see Supplementary Fig. 2).

Application of the method to observe ^13^C–^13^C correlations in the 18-kDa L99A mutant of T4 lysozyme (T4L L99A) is shown for arginine ^13^C^δ^–^13^C^γ^ in Fig. 2c, and for other side-chain correlations in Supplementary Figs. 3 and 4. Of importance is that (1) the NMR correlation spectrum in Fig. 2c is obtained in 37 min at 278 K, where the rotational correlation time of the 18-kDa T4L L99A mimics that of an ~30-kDa protein at room temperature, (2) six different ^13^C–^13^C correlation maps can be obtained in less than 12 min (see Supplementary Figs. 3, 4) and (3) a single uniformly [^2^H,^13^C] isotopically labelled sample could be used to characterise six side chains. The lack of directly bonded protons and absence of efficient relaxation pathways for aliphatic ^13^C in highly deuterated proteins dramatically reduces the ^13^C relaxation rates. For T4L L99A, the isoleucine ^13^C^δ1^ non-selective longitudinal relaxation rates, *R*_1_, range between 0.12 and 0.24 s^−1^ at 278 K and a field of 14.1 T (see Supplementary Table 1 and Supplementary Fig. 5). This necessitates longer recycling delays and fewer scans per unit time, and the lower gyromagnetic ratio of ^13^C compared with ^1^H leads to an intrinsic lower signal-to-noise. However, reduced relaxation rates also lead to small transverse relaxation rates, between 2.7 and 8.8 s^−1^ for isoleucine ^13^C^δ1^ in T4L L99A at 278 K and 14.1 T (see Supplementary Table 2 and Supplementary Fig. 7), which in turn lead to very sharp signals. For example, with the ^13^C-detected method, it became possible to observe sites in arginine side chains of T4L L99A that were not detectable in a ^1^H-detected equivalent experiment, even when using a highly optimised isotope-labelling scheme (Supplementary Fig. 6). Another striking advantage of ^13^C–^13^C correlation spectra compared with ^1^H–^13^C spectra is the substantially better chemical shift dispersion in the directly detected dimension, resulting in significantly better peak separation (see Supplementary Fig. 6 for a comparison). Moreover, as compared with ^1^H–^13^C spectra, the ^13^C–^13^C correlation maps directly provide the chemical shift of two aliphatic ^13^C that are both known to report on the structure and sampling of side chains^24,25^.

### Direct-detected ^13^C spectra of a 82-kDa protein

**Fig. 3:**
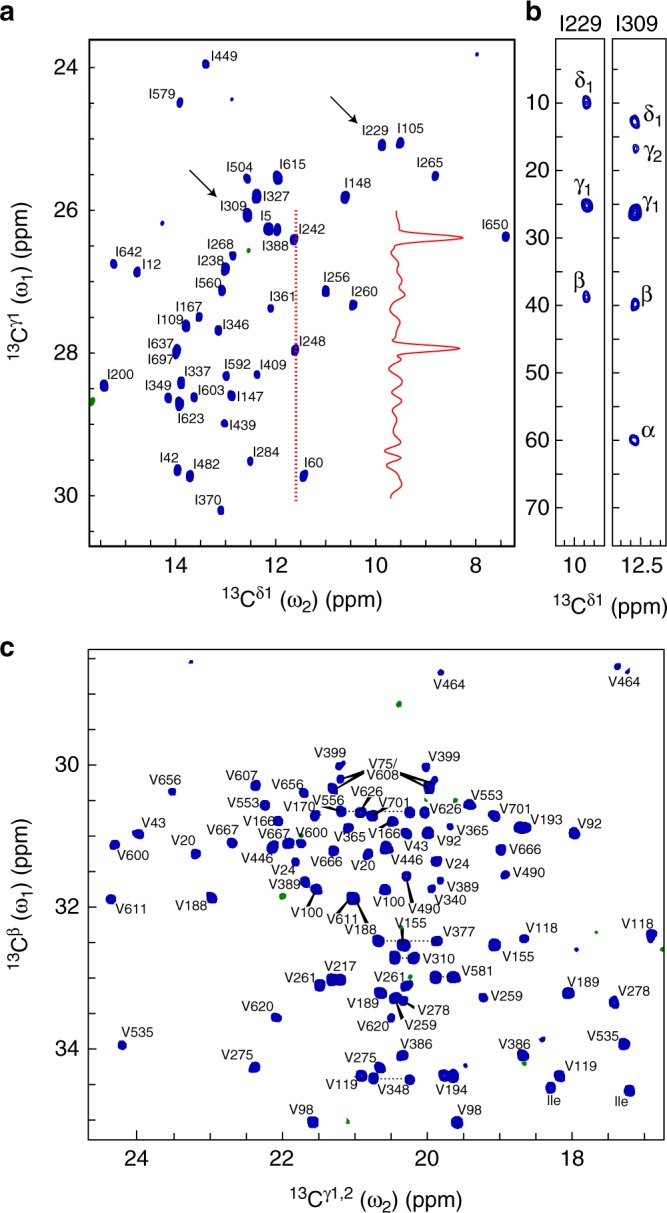
^13^C–^13^C side-chain correlation spectra of a large protein. **a** Isoleucine ^13^C^δ1^–^13^C^γ1^ and **c** valine ^13^C^γ^–^13^C^β^ correlation spectra of a 0.5 mM sample of the 82-kDa protein MSG. Each of the two spectra in **a** and **c** were recorded in 7.5 h at 18.8 T using a standard helium-cooled Bruker TCI radio-frequency probe. **b** Correlating the ^13^C^δ1^–^13^C^γ1^ of isoleucine with the rest of the side chain ^13^C for chemical shift assignment. Representative TOCSY strips from a three-dimensional ^13^C-detected CC-TOCSY experiment (see Supplementary Fig. 8) for I229 and I309 (marked with black arrows in **a**. Chemical shift assignments in **a** and **c** were obtained from (refs. ^26,27^) combined with the 3D-CC-TOCSY. Peaks labelled Ile in **c** are isoleucine ^13^C^γ2^–^13^C^β^ correlations. All experimental parameters are given in Supplementary Materials and Methods

The excellent spectra obtained on the medium-sized T4L L99A protein at low temperature and the favourable ^13^C transverse relaxation rates show that ^13^C-direct detection is ideally suited for characterising side chains in large proteins. Side-chain ^13^C–^13^C correlation maps were recorded for the significantly larger 82-kDa malate synthase G (MSG) protein^26^. Figure 3a and c shows the isoleucine ^13^C^δ1^–^13^C^γ1^ and valine ^13^C^γ^–^13^C^β^ correlation maps of MSG, respectively, where excellent chemical shift dispersion and resolution result in nearly no overlap of peaks even in this large and uniformly [^2^H,^13^C] isotopically labelled system. The slow transverse relaxation rate of aliphatic ^13^C in per-deuterated proteins means that the experiment shown in Fig. 2a easily can be extended to obtain numerous other NMR parameters reporting on motions, conformations and interactions. TOtal Correlated SpectroscopY (TOCSY) NMR experiments are typically used to aid side-chain chemical shift assignment^28^ of proteins and a couple of representative examples are shown in Fig. 3b for MSG, with details of the sequence given in Supplementary Fig. 8.

### Characterisation of millisecond dynamics

A full characterisation of the role of side chains requires an appreciation of their motions within the protein. Of substantial importance is that the method detailed above can be extended to allow quantification of side-chain motions across a wide range of timescales. Although many experiments will be possible using the new scheme, two examples, which together report on side-chain motions on timescales from nanoseconds to milliseconds, were chosen here to highlight the versatile applicability.

**Fig. 4:**
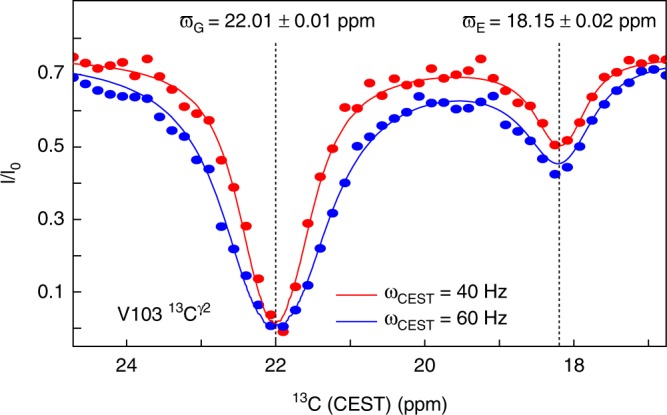
Quantifying side-chain motions on the millisecond timescale. CEST profiles to quantify millisecond chemical exchange of V103 ^13^C^γ2^ in T4L L99A at 278 K are shown. The circles represent experimentally obtained normalised intensities, and the lines are the result of a least-squares fit. Uncertainties in the reported *ϖ*_G_ and *ϖ*_E_ are obtained using the covariance method in the least-squares fit (see Materials and Methods for full details)

Protein dynamics and conformational exchange on the millisecond timescale have been shown to be important for the function of many proteins^29^ and the chemical exchange saturation transfers (CEST)^19,30^ NMR experiment, amongst others, allows a quantification of exchange events on this timescale. CEST experiments have recently been adapted for side chains in small proteins^14^, in a ^1^H-detected manner. Integrating CEST with the ^13^C-detected method described above (see Supplementary Fig. 9) allows quantification of side-chain conformational exchange in medium-to-large proteins between the ground state (G) and an excited state (E). Examples of CEST profiles are shown in Fig. 4 and Supplementary Fig. 9, where the conformational exchange of T4L L99A at 278 K is quantified by a ^13^C-detected CEST experiment. The calculated overall exchange rate, *k*_ex_ = 128 ± 18 s^−1^, and the population of the excited state, *p*_E_ = 1.15 ± 0.11% (see Supplementary Fig. 10), agree well with previous studies^11^ showing that reliable parameters are derived from ^13^C-detected CEST experiments. The chemical shifts of aliphatic ^13^C within the protein side chains report on the rotameric sampling of the side chain^24^, and the results of the CEST experiments in Fig. 4 also report on the rotameric sampling in the excited state. Specifically, from the chemical shifts obtained for V103 in the ground and excited states, ϖ_G_ and ϖ_E_, respectively, it can be calculated^24^ that the χ_1_ angle of V103 changes from a predominantly trans conformation (91% trans, 9% gauche-plus) in the ground state to a mainly gauche-minus conformation in the excited state (26% gauche-plus, 16% trans, 58% gauche-minus), which is in agreement with the existing structures of T4L L99A in the ground and the excited state^11^. Access to the ^13^C chemical shifts in the excited state via the ^13^C-detected CEST experiment also reveals that the V103 side chain is more dynamic in the excited state with a broader distribution of side-chain rotamers.

### Measuring long-range ^13^C–^13^C scalar couplings

**Fig. 5:**
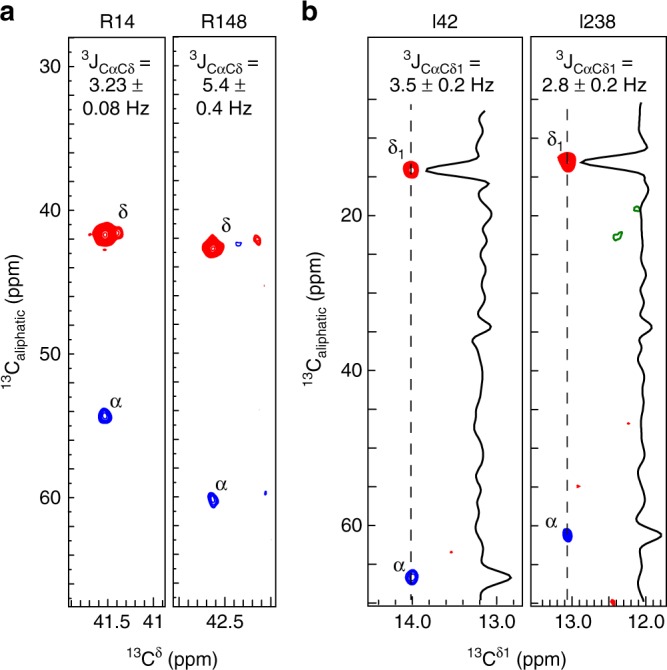
Quantifying long-range scalar ^13^C–^13^C couplings. **a** Two examples of strips used to quantify long-range ^13^C^δ^–^13^C^α^ scalar couplings in the arginine side chains of T4L L99A at 18.8 T and 278 K. The R14 side chain is flexible, while the R148 is rigid in agreement with relaxation measurements^6^. **b** Strips from a three-dimensional experiment to quantify long-range ^13^C^δ1^–^13^C^α^ scalar couplings in isoleucine side chains of the 82-kDa MSG at 14.1 T and 310 K. The I238 side chain is dynamic around the χ_2_ angle, as seen by the intermediate value of the ^3^*J*_CαCδ1_ coupling constant, whereas I42 is more rigid in a trans conformation. Positive contours are depicted as red, while negative contours are in blue. Values for ^3^*J*_CαCδ1_ in **a** and **b** are mean ± s.d.

The second application involves the measurement of three-bond ^13^C^δ^–^13^C^α^ scalar couplings^13^, ^3^*J*_CαCδ_, reporting on the conformational sampling of the side-chain χ_2_ dihedral angle on a timescale from nanoseconds to milliseconds. The three-bond scalar coupling ^3^*J*_CαCδ_ relates to the side-chain χ_2_ dihedral angle via a Karplus relationship, where large coupling constants are observed when the χ_2_ angle is in a trans conformation, and small couplings are observed when χ_2_ is in a gauche conformation. Intermediate values of the coupling constant are observed when the side chain is dynamic about the χ_2_ dihedral angle. Extending the ^13^C–^13^C correlation experiment to measure these couplings (see Supplementary Fig. 10) showed that the majority of the arginine side chains in T4L L99A are predominantly in a trans conformation around the χ_2_ angle (see Supplementary Table 3) in agreement with crystallographic data. For the two arginine side chains of T4L L99A shown in Fig. 5a, the intermediate value of the coupling constant for R14 shows that this side chain is flexible and dynamic around the χ_2_ angle, while the high value observed for the R148 side chain shows a rigid trans conformation. This is in good agreement with previous characterisations of the dynamics of the arginine side chains of T4L L99A^6,31,32^, as well as with the structure of T4L L99A, where R14 is on the surface and R148 is engaged in a salt bridge with D10. Long-range ^13^C^δ^–^13^C^α^ scalar couplings were also measured for isoleucine residues in the 82 -kDa MSG protein (Fig. 5b; Supplementary Table 4). Of the 44 isoleucine residues in MSG, ^13^C^δ^–^13^C^α^ scalar couplings were obtained for 32, allowing a quantification of the dynamics of these side chains. Access to both the ^13^C^δ1^ chemical shift and the ^13^C^δ1^–^13^C^α^ scalar coupling allows a full characterisation of the rotameric sampling about the χ_2_ angle. For example, a large ^13^C^δ1^ chemical shift and a small ^13^C^δ1^–^13^C^α^ coupling for I200 in MSG show that this residue is restrained in a rare gauche-plus state (see Supplementary Table 4)^25^, in agreement with the crystal structure of MSG.

A strategy to characterise side chains in proteins using ^13^C-direct-detected NMR has been developed. Applications of ^13^C-detection to macromolecules has so far mainly been employed to investigate intrinsically disordered regions^18,22^, where many experiments are now available to exploit the improved resolution of ^13^C compared with ^1^H. Existing methods to characterise side chains in medium-to-large proteins have been limited to methyl-bearing residues and require specific labelling. The presented method employs ^13^C-detection and capitalises on the distinct chemical shift profiles of side chains to specifically observe different residues in a single uniformly labelled sample. As such, it allows a more general characterisation of side chains for investigations in large proteins (~82 kDa) and for a quantification of the dynamics of these side chains. The slow transverse ^13^C relaxations rates in fully deuterated proteins enables a large range of NMR experiments to characterise side-chain structure, interactions and dynamics. A few examples of experiments integrated with the side-chain ^13^C–^13^C-direct-detected method are detailed above. Combined with recent developments of hardware aimed at ^13^C-direct-detected NMR spectroscopy, we envisage that the new method will be particularly useful for experimentally quantifying functional side chains, at atomic resolution, in medium-to-large proteins. Forthcoming applications of this suite of methods promise a burgeoning appreciation of the role side chains play in orchestrating protein function.

## Methods

### Sample preparations

Isotopically labelled T4 lysozyme mutant C54T/C97A/L99A (T4L L99A) was expressed and purified as described previously^33^. Briefly, a codon-optimised form of the gene containing the mutations L99A, C54A and C97A in a kanamycin-resistant, pET-29b vector was transformed into BL21 (DE3) chemically competent *E. coli* cells. A single colony was used to inoculate 5-mL culture, which was grown overnight at 37 °C. This was used to inoculate 50 mL of minimal M9 media made with ^2^H_2_O and supplemented with 1 g L^−1^ [^1^H,^15^N]-ammonium chloride as the sole nitrogen source. For the uniformly labelled [^2^H,^13^C,^15^N] sample, 2 g L^−1^ [^2^H, ^13^C]-glucose was added as the sole carbon source. For the [^1^H^13^C-Lys,Arg; ^2^H^12^C] isotopically labelled sample, 2 g L^−1^ [^2^H, ^12^C]-glucose was added to the media, and 0.15 g L^−1^ [^1^H^13^C]-L-lysine, and 0.15 g L^−1^ [^1^H,^13^C]-L-arginine was later added 1 h prior to induction. This culture was grown overnight at 37 °C and used to inoculate a final 2-L culture. The final culture was grown to OD_600_ ≈ 0.6 before a 16-h induction with 1 mM IPTG at 18 °C. The T4L L99A protein was initially purified by ion-exchange chromatography. After lysis by sonication in (50 mM NaPO_4_ (pH 6.5), 2 mM EDTA, 5 mg of DNAse1 (Sigma) and 1 cOmplete^TM^ Mini Protease Inhibitor Cocktail tablet (Sigma) per 50-mL lysate), the soluble fraction was loaded onto a 5-mL HiTrap SP Sepharose FF column (GE Healthcare) (50 mM NaPO_4_ (pH 6.5), 2 mM EDTA). Protein was eluted from the column using a gradient of 1 M NaCl and T4L99A eluted at ~300 mM NaCl. Pooled fractions were further purified by size-exclusion chromatography using a Superdex S75 gel filtration column (GE Healthcare) (50 mM NaPO_4_ (pH 5.5), 2 mM EDTA and 25 mM NaCl). Pooled fractions were buffer exchanged into the final NMR buffer (50 mM NaPO_4_ (pH 5.5), 2 mM EDTA, 25 mM NaCl and 2 mM NaN_3_). The NMR sample contained 1.4 mM protein in 95%/5% ^1^H_2_O/^2^H_2_O for the uniformly [^2^H^13^C^15^N] labelled sample and 1.5 mM protein in 100% ^2^H_2_O for the [^1^H^13^C-Lys,Arg; ^2^H^12^C] labelled sample.

Isotopically labelled MSG was produced as described previously^26^. The gene with a C-terminal hexahistidine tag in an kanamycin-resistant pET-28a vector was transformed into BL21 (DE3) chemically competent *E. coli* cells. A single colony was used to inoculate 5-mL culture, which was grown overnight at 37 °C. This was used to inoculate 50 mL of M9 minimal media made with ^2^H_2_O and supplemented with 1 g L^−1^ [^1^H,^15^N]-ammonium chloride and 2 g L^−1^ of [^2^H,^13^C]-glucose as the sole nitrogen and carbon sources, respectively. The pre-culture was used to inoculate 1 L of M9 media, which was grown at 37 °C to OD_600_≈0.45 before a 1-in-2 dilution to make the final 2-L culture volume. This final culture was grown to OD_600_ ≈ 0.45 before a > 16-h induction with 1 mM IPTG at 16 °C. The MSG protein was initially purified by affinity chromatography. After lysis by sonication (20 mM Tris, 300 mM NaCl, 20 mM 2-mercaptoethanol, 10 mg of DNase1 (Sigma), 10 mg of hen egg lysozyme (Sigma) and 1 cOmplete^TM^ Mini Protease Inhibitor Cocktail tablet (Sigma) per 50-mL lysate), the soluble fraction was loaded onto a HisTrap 5-mL HP column (GE Healthcare) (20 mM Tris, 300 mM NaCl and 20 mM 2-mercaptoethanol, pH 7.8). Protein was eluted from the column using a gradient of 250 mM imidazole in the same buffer and the MSG protein eluted at ~90 mM imidazole. Protein in pooled fractions was unfolded (20 mM Tris, 100 mM NaCl, 10 mM 2-mercaptoethanol and 6 M guanidium chloride, pH 7.8) for 1 h at room temperature to allow full exchange of amide protons. Protein was refolded by rapid dilution (~1:50) into ice-cold refolding buffer (20 mM Tris, 100 mM NaCl, 10 mM 2-mercaptoethanol, 5 mM MgSO_4_, 10% sucrose and 6 cOmplete^TM^ Mini Protease Inhibitor Cocktail tablets (Sigma) per litre buffer, pH 7.8). The protein was incubated, stirring at room temperature for 2 h before purification by affinity chromatography as described above. The pooled fractions were further purified by size-exclusion chromatography using a Superdex 200 5/150 gel filtration column (GE Healthcare) (20 mM NaPO_4_, 5 mM dithiothreitol) and the MSG eluted at ~75 mL. Overall, 20 mM MgCl_2_, 0.05% NaN_3_ and 10% ^2^H_2_O was added to the samples to make up the final NMR buffer.

### Database extractions

The ^13^C chemical shift distributions for arginine, isoleucine, valine, threonine, lysine and proline shown in Supplementary Fig. 1 were extracted from the BMRB database^20^ using the PACSY database^34^ and the provided API (https://github.com/uwbmrb/BMRB-API), which was embedded in an in-house written python script.

### NMR spectroscopy

All ^13^C-detected experiments were performed on a Bruker Avance II 600-MHz spectrometer using a ^13^C-optimised TXO coldprobe or a Bruker Avance HD 800-MHz spectrometer using an HCN inverse TCI coldprobe equipped with cooled ^1^H and ^13^C preamplifiers.

### NMR experiments on T4L L99A

The two-dimensional ^13^C–^13^C correlation spectra in Fig. 2c, Supplementary Figs. 3 and 4 were all recorded at a static magnetic field of 14.1 T and a temperature of 278 K. The pulse sequence used was the one shown in Fig. 2a and Supplementary Fig. 2. Deuterium ^2^H decoupling was achieved using a constant-wave (CW) field applied at 1.1 kHz, which in our hands gave significantly better decoupling than using composite decoupling schemes, e.g. WALTZ16^35^. During the INEPT transfers, the ^2^H decoupling field was centred on the frequency of the ^2^H bound to ^13^C_t_, while for indirect chemical shift evolution, the ^2^H decoupling field was centred on the frequency of the ^2^H bound to ^13^C_p_. For example, for arginine ^13^C^δ^–^13^C^γ^, the ^2^H decoupling field was centred at 1.55 ppm (^2^H^γ^) during the indirect chemical shift evolution and at 3.10 ppm elsewhere. Frequency-selective 90° (180°) pulses were applied with RE-BURP (E-BURP)^36^ shapes. Spectra shown to the left (right) in Supplementary Figs. 2 and 3 were recorded with 10 (33) complex points in the indirect dimension, 4 scans per transient and a recovery delay of 4 s, leading to a total acquisition time of 12 min (38 min) per spectrum.

The ^13^C-detected CEST experiments were recorded at 14.1 T using the pulse sequence shown in Supplementary Fig. 9. CEST experiments to characterise valine residues were recorded with 22 complex points in the ^13^C^β^ dimension (sweep width of 800 Hz) and 50 CEST saturation points between −600 and 600 Hz. Eight scans were recorded per transient and a recovery delay of 4 s was used, leading to a total acquisition time of 40 h. CEST experiments to characterise arginine and threonine residues were recorded with 30 complex points in the ^13^C^γ^ dimension (sweep width of 1136 Hz) and 42 CEST saturation points between −300 and 300 Hz. Sixteen scans were recorded per transient and a recovery delay of 4.4 s was used, leading to a total acquisition time of 98 h. The CEST field, ω_CEST_, and its inhomogeneity was obtained, as described by Guenneugues et al.^37^, using a 20 mM sample of [U-^2^H,^13^C]-isoleucine. Uncertainties in *I*/*I*_0_ were estimated from duplicate measurements.

The 3D long-range ^13^C–^13^C scalar coupling experiment shown in Fig. 5a was recorded at 18.8 T and obtained using the pulse sequence shown in Supplementary Fig. 11. The spectrum was recorded with 42 complex points in the indirect ^13^C^γ1^ constant-time period (*t*_2_), 24 complex points in the indirect *t*_1_ period, 8 scans per transient and a recovery delay of 4 s, leading to a total acquisition time of 75 h. Further experimental details are given in the legend of Supplementary Fig. 11.

### NMR experiments on MSG

The two-dimensional ^13^C–^13^C correlation spectra in Fig. 1e and S8 were recorded at a static magnetic field of 18.8 T and a temperature of 310 K. The pulse sequence used was the one shown in Fig. 2a and Supplementary Fig. 2. The two spectra in Fig. 3 were recorded with 43 complex points in the indirect constant-time dimension, 16 scans per transient and a recovery delay of 10 s, leading to a total acquisition time of 7.5 h per spectrum.

The 3D ^13^C–^13^C-TOCSY spectrum shown in Fig. 3b was recorded using the pulse sequence described in Supplementary Fig. 8. The spectrum was recorded with 54 complex points in the indirect ^13^C^γ1^ constant-time period (*t*_2_), 24 complex points in the indirect *t*_1_ period, 4 scans per transient and a recovery delay of 10 s, leading to a total acquisition time of 120 h. Further experimental details are given in the legend to Supplementary Fig. 8.

The 3D long-range ^13^C–^13^C scalar coupling experiment shown in Fig. 5b was obtained using the pulse sequence shown in Supplementary Fig. 11. The spectrum was recorded with 42 complex points in the indirect ^13^C^γ1^ constant-time period (*t*_2_), 27 complex points in the indirect *t*_1_ period, 8 scans per transient and a recovery delay of 7.8 s, leading to a total acquisition time of 160 h. Further experimental details are given in the legend to Supplementary Fig. 11.

### Chemical shift assignments

The side-chain chemical shift assignment of T4L L99A was obtained based on the assignments published previously^6,11^ combined with ^1^H and ^13^C-detected CC-TOCSY experiments and the ^1^H and ^13^C-detected long-range ^3^*J*_CαCδ_ experiments. The stereospecific assignment of the valine ^13^C^γ^ was taken from ref. ^38^.

The chemical shift assignment of the MSG valine ^13^C^γ^–^13^C^β^ correlation spectrum was transferred from a previously published assignment^26,27^, that in turn was based on ^1^H-detected experiments. The chemical shift assignment of the isoleucine ^13^C^δ1^–^13^C^γ1^ spectrum was obtained by a combination of a previous assignment^26,27^ and the ^13^C-detected TOCSY experiment (Fig. 3b; Supplementary Fig. 8) and the ^13^C-detected long-range ^3^*J*_CαCδ_ experiment (Fig. 5b; Supplementary Fig. 11).

### Data analysis

All NMR spectra were processed using nmrPipe^39^ and initially analysed using NMRFAM-Sparky^40^ or CCPN^41^. Peak intensities in experiments reporting on relaxation rates and CEST, Fig. 4 and Supplementary Figs. 5, 7, 9 and 10, were obtained using FuDA^42,43^.

Long-range scalar couplings were derived by first obtaining the peak heights of the peaks corresponding to the diagonal peak, *I*_d_ *=* *I*(^13^C^δ^–^13^C^γ^–^13^C^δ^) and the cross-peak *I*_c_ *=* *I*(^13^C^α^–^13^C^γ^–^13^C^δ^) using the inbuilt tools of NMRFAM-Sparky and CCPN. The long-range scalar coupling was then calculated according to^4^ *I*_c_/*I*_d_ = tan^2^(2π*J*_CC_*T*_J_), where *J*_CC_ is the three-bond scalar coupling and *T*_J_ is the coupling evolution delay in the pulse sequence in Supplementary Fig. 11. As described previously^13^, passive couplings cancel by taking the ratio of *I*_c_ and *I*_d_, such that this ratio only reports on the long-range coupling. For the ^1^H-detected experiments, the error was determined as the root-mean-square deviation of two experiments. For the other long-range scalar coupling experiments, errors in the obtained *I*_c_ and *I*_d_ were estimated from RMSD of the spectral region, where no peaks were observed. The uncertainty of the calculated ^3^*J*_CC_ was estimated using a Monte-Carlo procedure to propagate the errors from the intensities.

Carbon ^13^C-detected CEST experiments were analysed using in-house written software in python using the LMFIT^44^ module for least-squares minimisation of the target function

1 $$\chi ^2({\mathbf{p}}) = \mathop {\sum }\limits_i \left( {\tilde I_{{\mathrm{obs}},i} - \tilde I_{{\mathrm{calc}},i}({\mathbf{p}})} \right)^2/\sigma ^2$$

where $\tilde I_{{\mathrm{obs}},i}$ and σ are the experimentally obtained normalised intensities $\left( {\tilde I = I/I_0} \right)$ and their uncertainty, respectively. The sum is over the different CEST saturation points and CEST field strengths, ω_CEST_. $\tilde I_{{\mathrm{calc}},i}$ are the calculated normalised intentities calculated as a function of the model parameters **p**. Briefly, calculated intensities were obtained by evolving the spin system according to the Liouvillian described previously^14,19,45,46^, also taking into account the one-bond ^13^C–^13^C scalar coupling, which generally leads to a simple line broadening of the CEST profiles. An inhomogeneity of the saturation field of 5% was used. The model parameters consist of the chemical shifts of the ground and excited states, *ϖ*_G_ and *ϖ*_E_, respectively, the longitudinal relaxation rate that was assumed to be identical in the ground and excited state, *R*_1G_ = *R*_1E_ = *R*_1_ and the transverse relaxation rate in the ground and excited states, *R*_1G_, *R*_2E_. For the analysis of R125, it was imposed that *R*_2G_ = *R*_2E_, since the data did not contain enough information to determine *R*_2E_; for other fits, both *R*_2G_ and *R*_2E_ were determined.

The overall exchange range, *k*_ex_, and the population of the excited state, *p*_E_, were treated as global parameters. Uncertainties of the obtained residue-specific parameters were estimated using the covariance method^47^, as implemented in LMFIT. The uncertainties of *k*_ex_ and *p*_E_ were estimated by performing a grid search (see Supplementary Fig. 10).

### Reporting summary

Further information on research design is available in the Nature Research Reporting Summary linked to this article.

## Supplementary information

Supplementary Information

Peer Review

Reporting Summary

Source data

## Data Availability

The data that support the findings of this study and the pulse programmes (Bruker) are available from the corresponding author upon reasonable request. The source data underlying Figs. 1, 4 and Supplementary Figs. 1, 5, 7 and 9 are provided as a Source Data file.

## Code availability

The pulse programmes (sequences) are available for Bruker spectrometers from the corresponding author upon request.
