# Supplementary material for: Characterising side chains in large proteins by protonless 13C-detected NMR spectroscopy
Source: Nat Commun. 2019 Apr 15;10:1747. doi: 10.1038/s41467-019-09743-4 (PMC6465260; doi:10.1038/s41467-019-09743-4)
Supplement: Supplementary file 1 — Supplementary Information [file 41467_2019_9743_MOESM1_ESM.pdf]

SUPPLEMENTARY INFORMATION for

Characterising side chains in large proteins by protonless ^{13}C-detected NMR spectroscopy

Pritchard and Hansen

Supplementary Figures

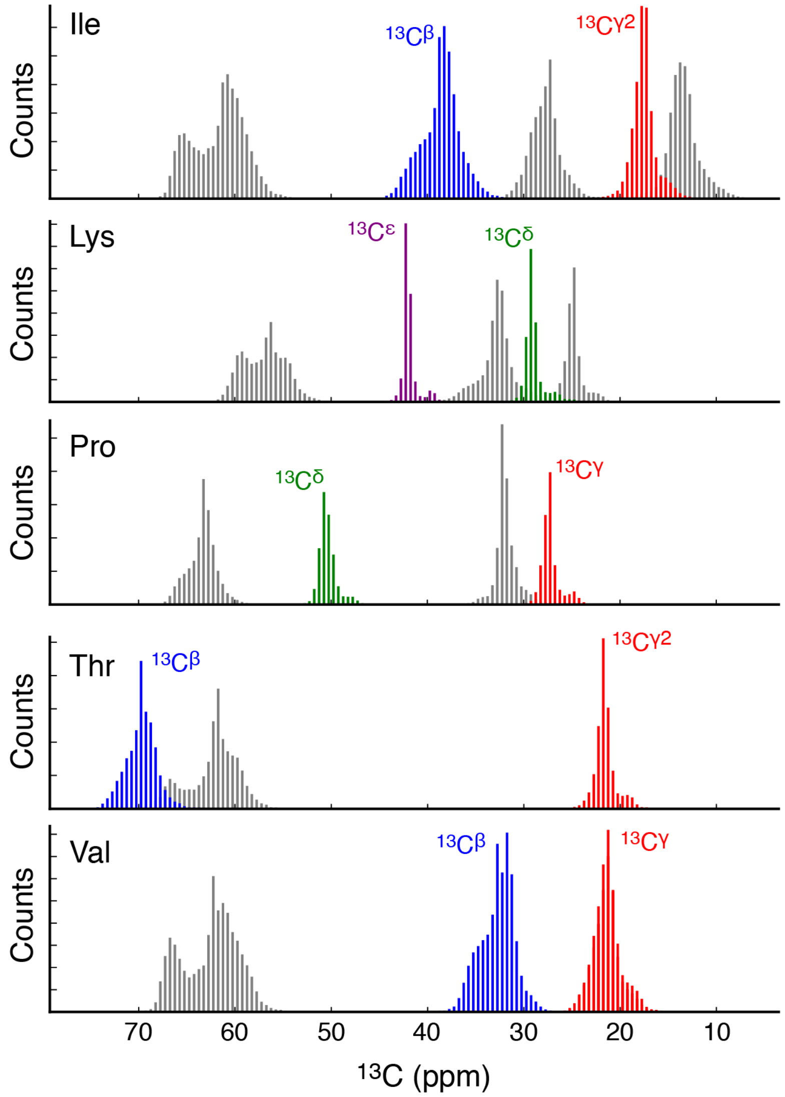

Supplementary Figure 1: Side-chain ^{13}C chemical shift distributions obtained from the BMRB[1] databank. The nuclei probed in the ^{13}C-^{13}C correlation maps are coloured according to their type; blue: $^{13}C^{\beta}$, red: $^{13}C^{\gamma}$, green: $^{13}C^{\delta}$ and purple: $^{13}C^{\varepsilon}$. Passive nuclei, that is, those not observed in the two-dimensional ^{13}C-^{13}C correlation spectra are coloured grey. It is noted that the seven side-chain experiments {Arg $^{13}C^{\delta}$-$^{13}C^{\gamma}$, Ile $^{13}C^{\delta 1}$-$^{13}C^{\gamma 1}$, Ile $^{13}C^{\gamma 2}$-$^{13}C^{\beta}$, Lys $^{13}C^{\delta}$-$^{13}C^{\varepsilon}$, Pro $^{13}C^{\delta}$-$^{13}C^{\gamma}$, Thr $^{13}C^{\gamma 2}$-$^{13}C^{\beta}$, Val $^{13}C^{\gamma}$-$^{13}C^{\beta}$} performed based on the distributions above and in Fig. 1 are generally selective for the nuclei and residue type chosen. Exceptions are the Valine $^{13}C^{\gamma}$-$^{13}C^{\beta}$ and Isoleucine $^{13}C^{\gamma 2}$-$^{13}C^{\beta}$ experiments, as well as Arginine $^{13}C^{\delta 1}$-$^{13}C^{\gamma 1}$ and Lysine $^{13}C^{\varepsilon}$-$^{13}C^{\delta}$, where signals from one experiment may appear in the other (see Figs. 2c and 3c).

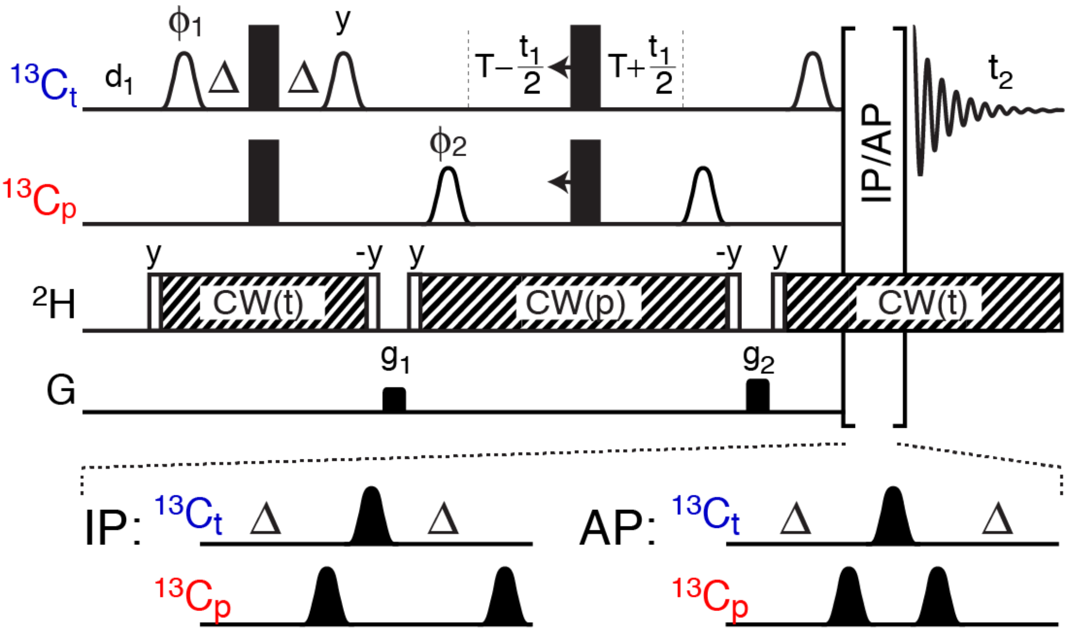

Supplementary Figure 2: ^{13}C-^{13}C side-chain correlation pulse sequence. Core element, pulse scheme, derived to obtain two-dimensional $^{13}C_t$-$^{13}C_p$ correlation spectra of side chains in per-deuterated proteins. Black bars represent 180° non-selective pulses and open bell-shaped pulses represent frequency-selective 90° pulses. The ^{13}C carrier is placed in the middle of the $^{13}C_t$ region, with the exception of the indirect chemical shift evolution period, where the carrier is placed in the middle of the $^{13}C_p$ region. As such, all frequency-selective pulses are applied on-resonance, except for the two 180° $^{13}C_p$ pulses in the IPAP block that are frequency-shifted by a linear phase modulation (see Table S5). The following delays are used: $\Delta = 1/(4J_{CC}) \approx 7.1$ ms, $T = 1/(2J_{CC}) \approx 14.1$ ms (See Table S5). The frequency selective 90° pulses are applied with an E-BURP[2] shape with a length of 2 ms at 18.8 T. Gradients of 1 ms are represented by black rectangles and applied with strength of g_1: 3.7 G/cm, g_2: 9.1 G/cm. The phase cycle is ϕ_1: 2(x), 2($-x$); ϕ_2: x, $-x$; ϕ_{rec}: x, $-x$, $-x$, x. Frequency discrimination is obtained by States-TPPI[3] of the phase ϕ_2. Deuterium decoupling, ^2H, is achieved using a constant wave scheme applied at 1.1 kHz with x-phase. Flanking 90° pulses are applied before and after the decoupling to obtain a more stable lock-signal. The carrier of ^2H is placed at the ^2H_t hydrogen chemical shift (CW(t)) during the INEPTs where magnetisation is transverse with respect to $^{13}C_t$ and at the ^2H_p hydrogen chemical shift (CW(p)) during the indirect chemical shift evolution, where magnetisation is transverse with respect to $^{13}C_p$ (Supplementary Table 5). The lock is held during the first 1/3 of d_1 and sampled during the last 2/3 of d_1 to ensure stability of the locking. Optimal sensitivity per unit time is achieved with $d_1 \sim 1.26/R_1$. The IPAP block is shown below the pulse sequence, where the two sub-spectra required for IPAP are acquired before the t_1 loop. Virtual decoupling using IPAP leads to half the number of peaks in the spectrum and an improvement in signal-to-noise by a factor of $\sqrt{2}$ compared to no decoupling.

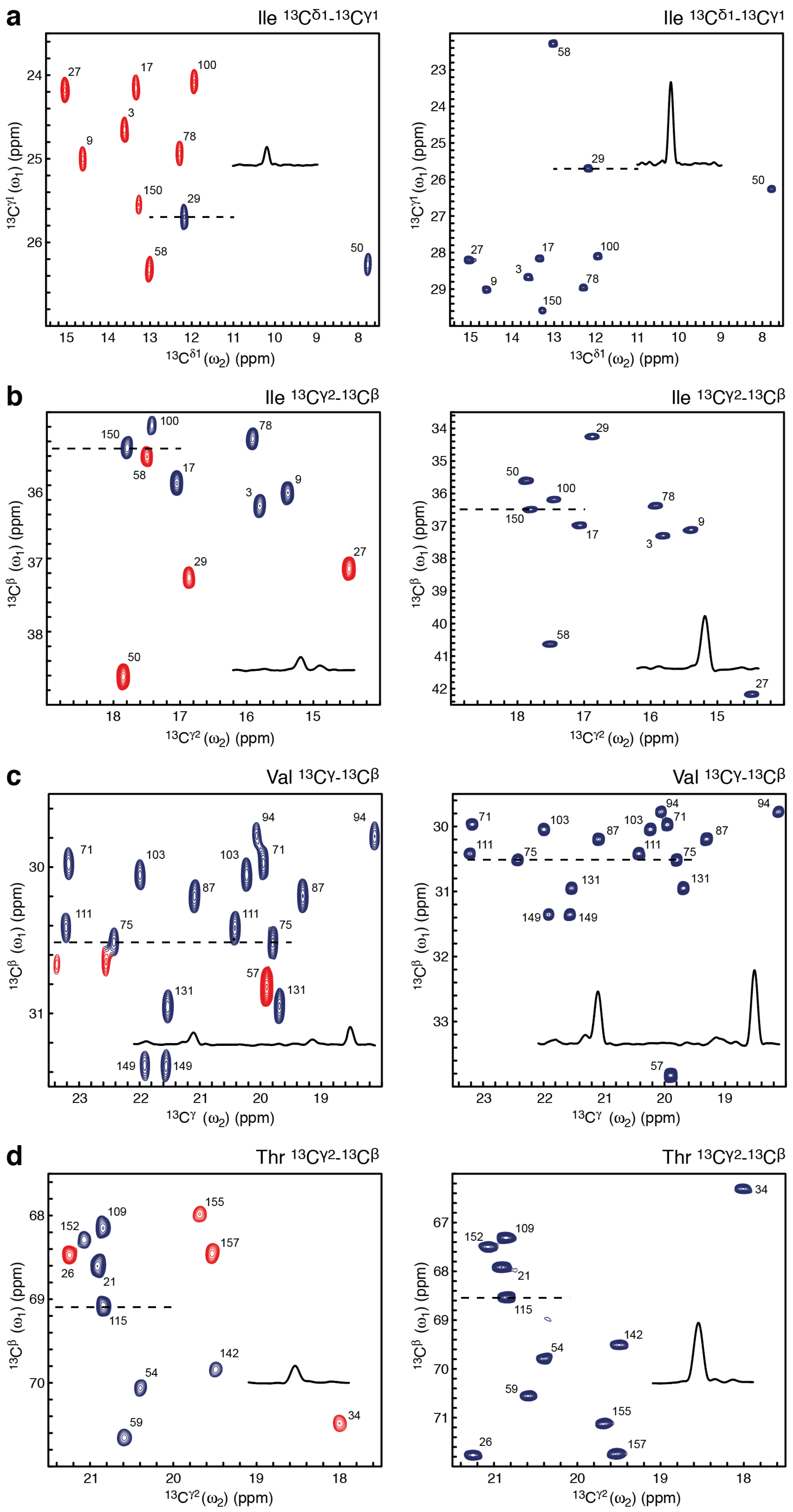

a
Ile $^{13}C^{\delta1}$-$^{13}C^{\gamma1}$
$^{13}C^{\gamma1}$ (ω_1) (ppm)
$^{13}C^{\delta1}$ (ω_2) (ppm)
b
Ile $^{13}C^{\gamma2}$-$^{13}C^{\beta}$
$^{13}C^{\beta}$ (ω_1) (ppm)
$^{13}C^{\gamma2}$ (ω_2) (ppm)
c
Val $^{13}C^{\gamma}$-$^{13}C^{\beta}$
$^{13}C^{\beta}$ (ω_1) (ppm)
$^{13}C^{\gamma}$ (ω_2) (ppm)
d
Thr $^{13}C^{\gamma2}$-$^{13}C^{\beta}$
$^{13}C^{\beta}$ (ω_1) (ppm)
$^{13}C^{\gamma2}$ (ω_2) (ppm)

Supplementary Figure 3: ^{13}C-^{13}C correlation NMR spectra of methyl-bearing side chains in T4L99A. All spectra were recorded using a 1.4 mM sample of T4L L99A at a static magnetic field of 14.1 T and at a temperature of 278 K using the pulse sequence shown in Fig. 2 and Supplementary Figure 2. Spectra in the left column were recorded with a total acquisition time of 12 min with a minimal sweep width in the indirect dimension (4 ppm for ***a***,***b***,***d*** and 3 ppm for ***c***), such that some peaks (in red) are folded. Spectra in the right column were recorded for 38 min using a full sweep width (10 ppm) in the indirect dimension. Chemical shift assignments obtained from ^{13}C-^{13}C TOCSY experiments (Supplementary Figure 8) and the previous assignment of T4L L99A[4] are indicated with residue numbers. The one-dimensional spectra shown are slices taken out at the dashed lines to show the signal-to-noise. ***a*** Isoleucine $^{13}C^{\delta1}$-$^{13}C^{\gamma1}$ correlation spectra, ***b*** isoleucine $^{13}C^{\gamma2}$-$^{13}C^{\beta}$ correlation spectra, ***c*** valine $^{13}C^{\gamma}$-$^{13}C^{\beta}$ correlation spectra, and ***d*** threonine $^{13}C^{\gamma2}$-$^{13}C^{\beta}$.

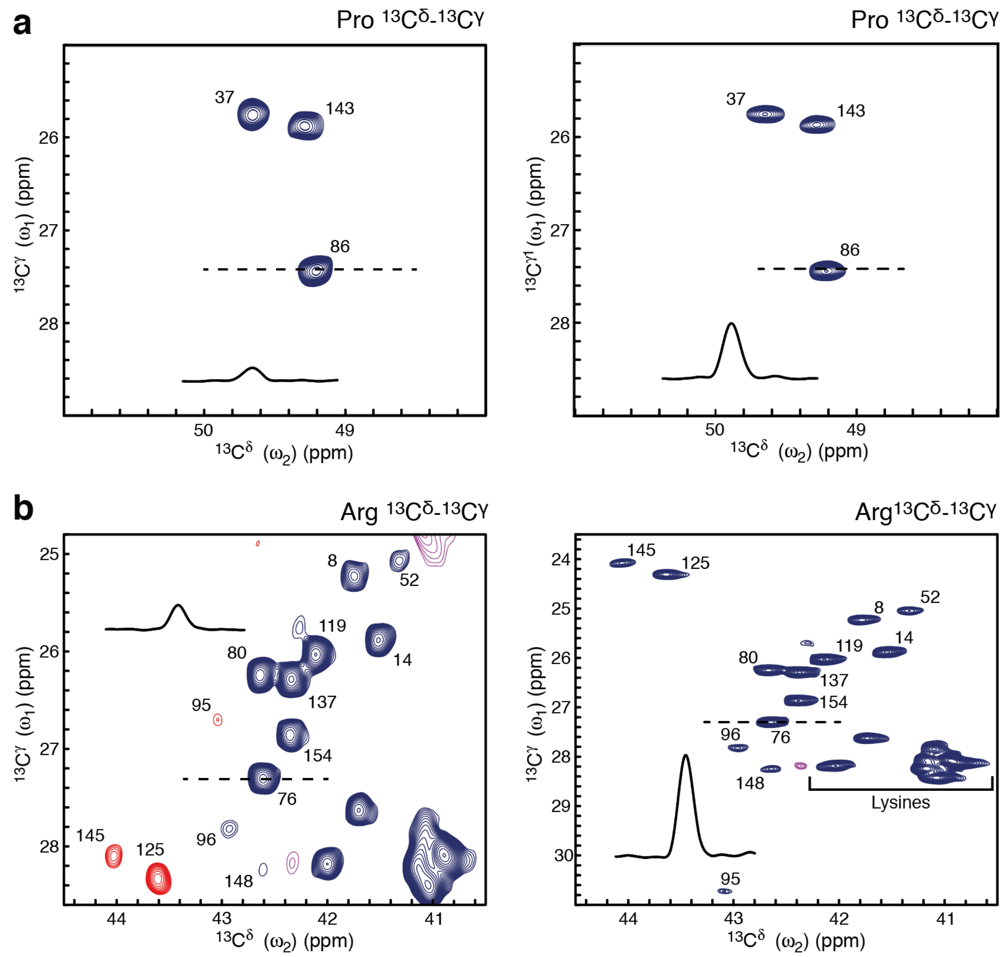

Supplementary Figure 4: ^{13}C-^{13}C correlation NMR spectra of non methyl-bearing side chains in T4L99A. All spectra were recorded using a 1.4 mM sample of T4L L99A at a static magnetic field of 14.1 T and at a temperature of 278 K using the pulse sequence shown in Fig. 2 and Supplementary Figure 2. Spectra in the left column were recorded with a total acquisition time of 12 min with minimal sweep width (4 ppm) in the indirect dimension, such that some peaks (in red) are folded. Spectra in the right column were recorded for 38 min using a full sweep width (10 ppm) in the indirect dimension. Chemical shift assignments are indicated with residue numbers as obtained from ^{13}C-^{13}C TOCSY experiments (Supplementary Figure 8) and the previous assignment of T4L L99A[4]. The one-dimensional spectra shown are slices taken out at the dashed lines to show the signal-to-noise. ***a*** Proline $^{13}C^{\delta}$-$^{13}C^{\gamma}$ correlation spectra, ***b*** arginine $^{13}C^{\delta}$-$^{13}C^{\gamma}$ correlation spectra.

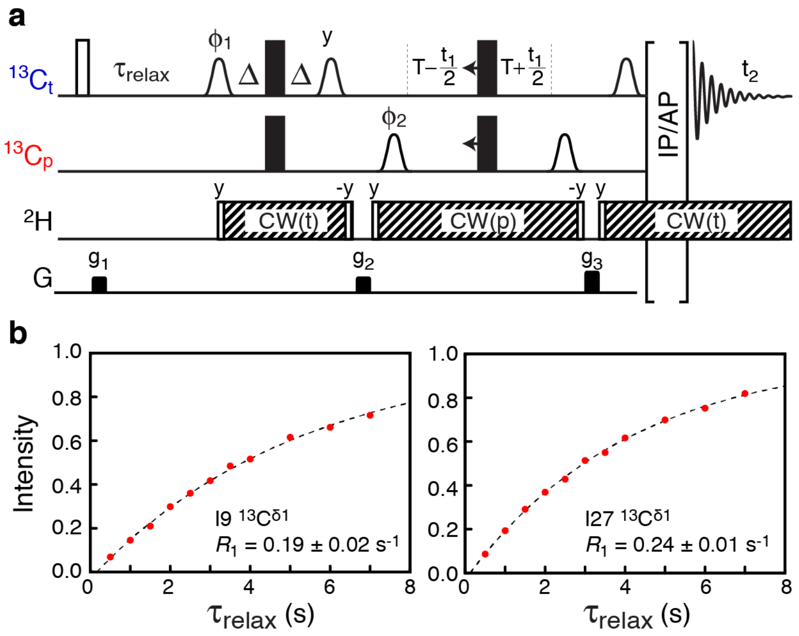

Supplementary Figure 5: Measurements of longitudinal R_1 rates. ***a*** Pulse scheme derived for measuring non-selective longitudinal ^{13}C relaxation rates. Black (open) bars represent 180° (90°) non-selective pulses and open bell-shaped pulses represent frequency-selective 90° pulses. The following delays are used: $\Delta = 1/(4J_{CC}) = 7.1$ ms, $T = 1/(2J_{CC}) = 14.1$ ms, and τ_{relax} in the range from 0.01 s to 8 s. The frequency selective 90° pulses are applied with an E-BURP[2] shape with a length of 2 ms at 18.8 T. Gradients of 1 ms are represented by black rectangles and applied with strength of g_1: 1.2 G/cm, g_2: 3.7 G/cm, g_3: 9.1 G/cm. The phase cycle is ϕ_1: 2(x), 2(-x); ϕ_2: x, -x; ϕ_{rec}: x, -x, -x, x. Frequency discrimination is obtained by States-TPPI[3] of the phase ϕ_2. Deuterium, ^{2}H, decoupling is performed as described in Supplementary Figure 2. Residual longitudinal magnetisation is initially purged by the 90° – g_1 element, such that recovery curves start at approximately zero intensity. These recovery curves can therefore directly be used to judge the recycling delay needed for other experiments, such as Supplementary Figure 2. Spectra were recorded in an interleaved manner with the two IPAP sub-spectra recorded as the inner loop, τ_{relax} as the middle loop, and the t_1 array as the outer and last loop. ***b*** Examples of relaxation decay curves for two representative isoleucine side chains in T4L L99A. The red filled circles are experimentally derived normalised intensities, where uncertainties of the intensities are within the size of the circles. The dashed line is a least-squares fit of the function $I(t) = I_\infty - (I_\infty - I_0)\exp(-R_1 t)$ to the data shown, where I_0 is the intensity at $t = 0$ ($I_0 \approx 0$) and I^{∞} is the equilibrium intensity approached for $t \longrightarrow \infty$. Results for all isoleucine side chains of T4L L99A are shown in Supplementary Table 1. For the T4L L99A protein studied here an interscan delay between 5 s and 10 s results in the maximum sensitivity per unit time.

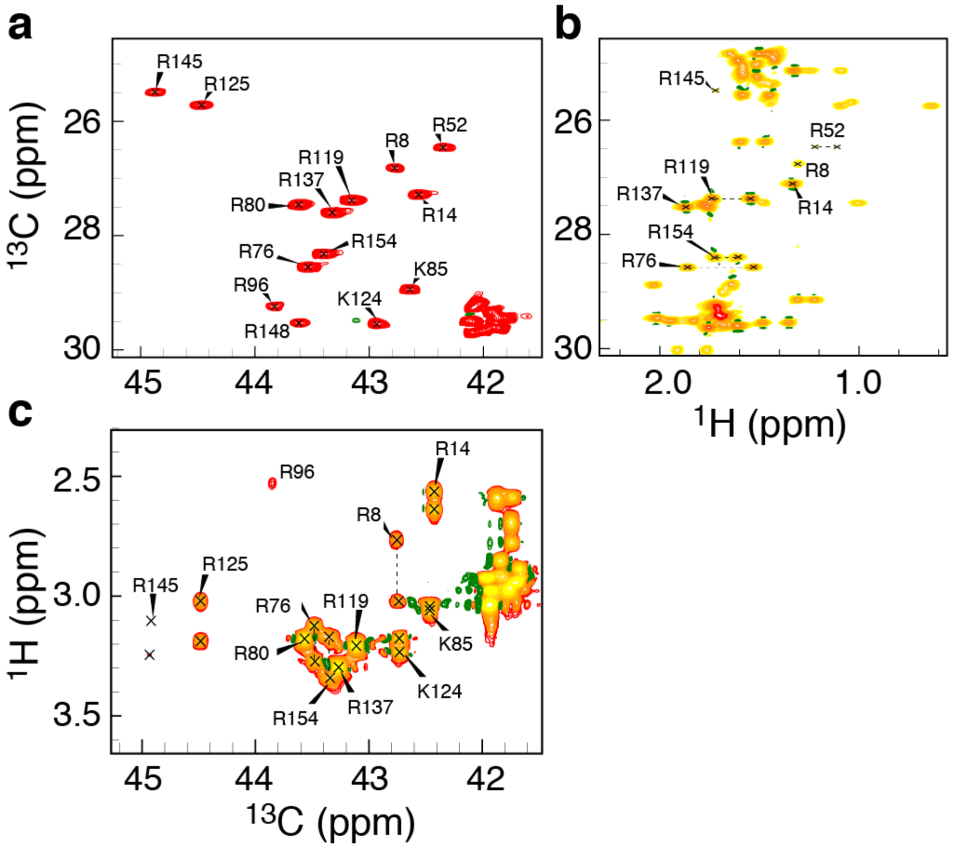

Supplementary Figure 6: Comparison of ^{13}C-^{13}C correlation maps and ^{1}H-^{13}C constant time HSQC. ***a*** A ^{13}C-^{13}C correlation spectrum of uniformly labelled [U-^{2}H^{13}C ^{15}N]-T4L L99A with focus on the arginine $^{13}C^{\delta}$-$^{13}C^{\gamma}$ recorded at 298 K and 18.8 T (1.4 mM; 38 min). Positive contours are depicted in red while negative contours are in green. ***b*** and ***c*** Excerpts of a constant time ^{1}H-^{13}C HSQC (1.5 mM; 67 min) of a selectively labelled [U-^{2}H^{12}C; Arg/Lys ^{1}H^{13}C^{15}N]-T4L L99A recorded at 298 K and 18.8 T. Positive contours are depicted with a yellow-to-red gradient to enhance visualisation and negative contours are in green. Despite having used a highly-optimised labelling scheme, a significant number of peaks could not be assigned due to low intensity and/or overlap in the constant-time ^{1}H-^{13}C HSQC.

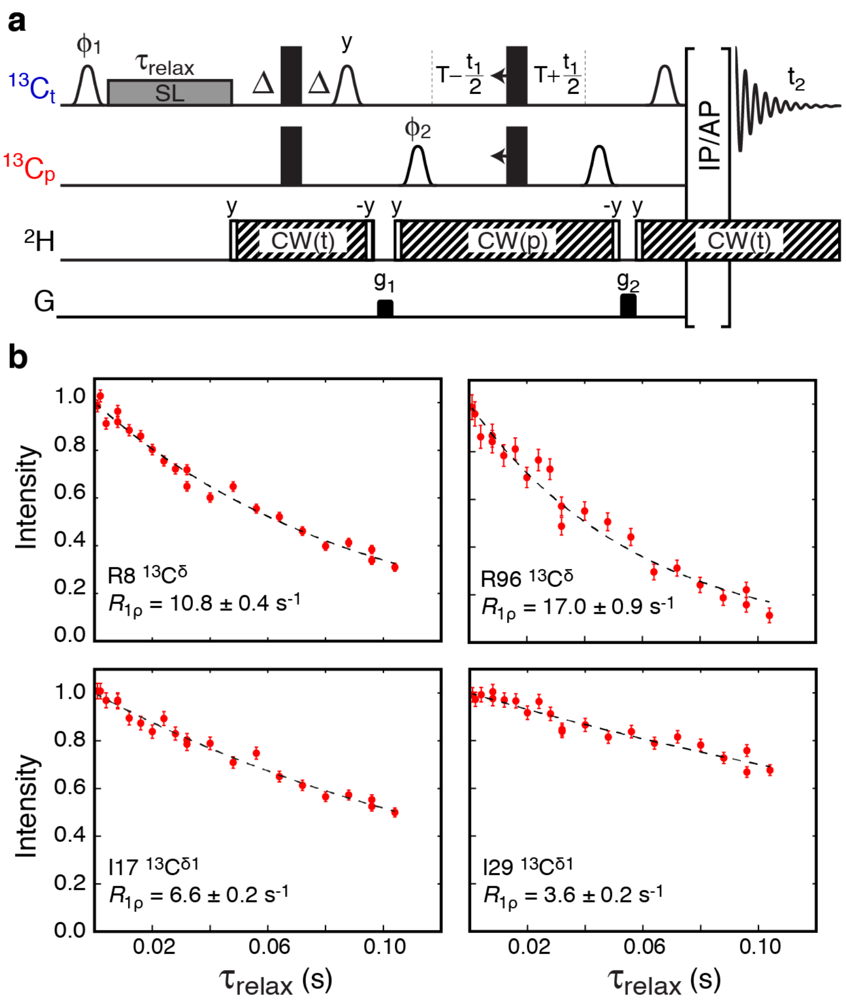

Supplementary Figure 7: Measurements of transverse $R_{1\rho}$ rates. ***a*** Pulse scheme derived for measuring the transverse ^{13}C relaxation rates in the rotating frame[5]. Black bars represent 180° non-selective pulses and open bell-shaped pulses represent frequency-selective 90° pulses. The following delays are used: $\Delta = 1/(4J_{CC}) \approx 7.1$ ms, $T = 1/(2J_{CC}) \approx 14.1$ ms. The frequency selective 90° pulses are applied with an E-BURP[2] shape with a length of 2 ms at 18.8 T. Deuterium, ^{2}H, decoupling is achieved with a continuous wave at a field of 1.1 kHz and centered at 0.65 ppm (3.1 ppm) during the INEPT and IPAP block, and at 1.25 ppm (1.55 ppm) during chemical shift evolution for isoleucine (arginine). IPAP[6] and post-processing are used as described above in Fig. 2 and Supplementary Figure 2. Gradients of 1 ms are represented by black rectangles and applied with strength of g_1: 3.7 G/cm, g_2: 9.1 G/cm. The phase cycle is ϕ_1: 2(x), 2(-x); ϕ_2: x, -x; ϕ_{rec}: x, -x, -x, x. Frequency discrimination is obtained by States-TPPI[3] of the phase ϕ_2. Spectra are recorded in an interleaved manner with the two IPAP sub-spectra recorded as the inner loop, τ_{relax} as the middle loop, and the t_1 array as the outer and last loop. ***b*** Example of a relaxation decay for two representative arginine side chains and two isoleucine side chains in T4L L99A. Red circles are experimentally obtained intensities, vertical bars represent the uncertainty of the obtained intensities calculated as the standard deviation from multiple measurements, and the dashed black lines are fits of a single exponential decay to the experimentally obtained intensities.

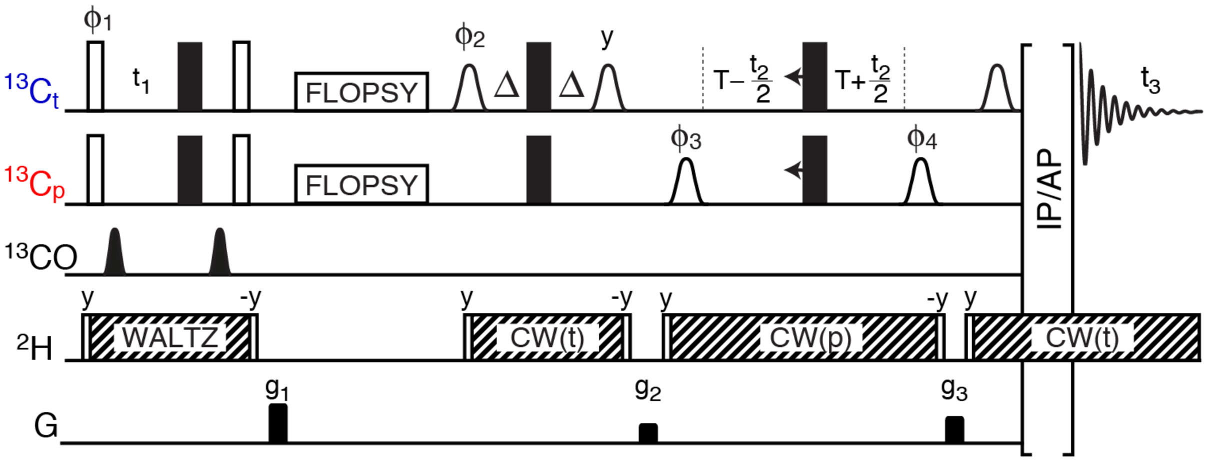

Supplementary Figure 8: Three-dimensional ^{13}C-detected TOCSY experiments for chemical shift assignments. Pulse scheme derived for correlating the aliphatic ^{13}C of the side chain with the terminal ($^{13}C_t$) and the penultimate ^{13}C ($^{13}C_p$) (see main text). Black bars represent 180° non-selective pulses and open (black) bell-shaped pulses represent frequency-selective 90° (180°) pulses. The following delays are used: $\Delta = 1/(4J_{CC})$= 7.1 ms, $T = 1/(2J_{CC})$ = 14.1 ms. The frequency selective 90° pulses are applied with an E-BURP[2] shape with a length of 2 ms at 18.8 T and the selective ^{13}CO decoupling pulses are applied with a Seduce1 shape[9] during t_1. The second ^{13}CO selective pulse during t_1 provides a Bloch–Siegert compensation for the first ^{13}CO pulse. Isotropic mixing is achieved with a FLOPSY-8[10] scheme applied at a field strength of 7.8 kHz for 18 ms and centered at 42 ppm. Deuterium, ^{2}H, decoupling is achieved with a waltz-16 scheme[11] during the first chemical shift evolution, and with a continuous wave at a field of 1.1 kHz for the rest of the sequence. The ^{13}C carrier is placed at 42 ppm during t_1 and isotropic FLOPSY mixing and moved to $^{13}C_t$ and $^{13}C_p$ during the rest of sequence. The ^{2}H carrier is placed at 2.75 ppm during waltz-16, at 0.65 ppm (3.1 ppm) during the INEPT and IPAP block, and at 1.25 ppm (1.55 ppm) during t_2 chemical shift evolution for isoleucine (arginine). IPAP[6] and post-processing are used for virtual decoupling of $^{13}C_t$ and $^{13}C_p$ during acquisition as described in Fig. 2 and Supplementary Figure 2. Gradients of 1 ms are represented by black rectangles and applied with strength of g_1: 17.7 G/cm, g_2: 3.7 G/cm, g_3: 9.1 G/cm. The phase cycle is ϕ_2: 2(x), 2(-x); ϕ_3: x, -x; ϕ_{rec}: x, -x, -x, x. Frequency discrimination is obtained by States-TPPI[3] of the phases ϕ_1 and ϕ_4 for the two indirect dimensions, respectively. Chemical shift evolution during t_1 is non-constant time, while chemical shift evolution during t_2 is recorded in a constant time manner.

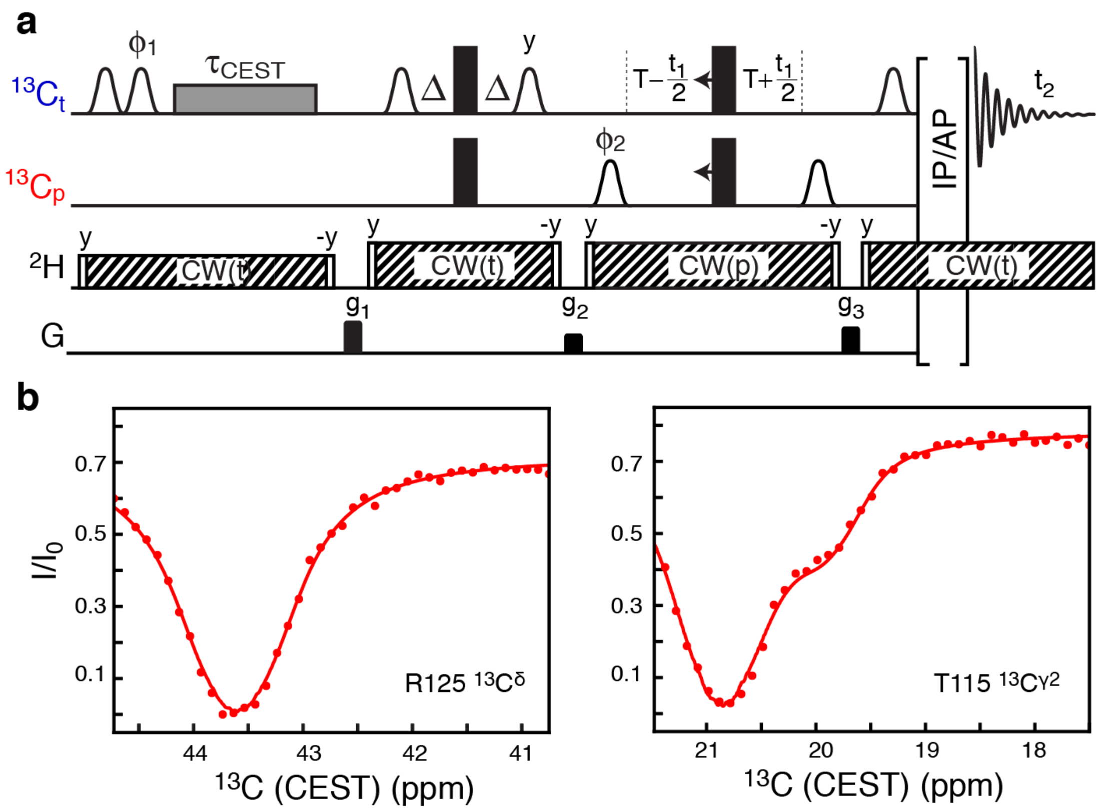

Supplementary Figure 9: Measurements of conformational exchange using Chemical Exchange Saturation Transfer.[12] ***a*** Pulse sequence derived to obtain ^{13}C CEST profiles. Black bars represent 180° non-selective pulses and open bell-shaped pulses represent frequency-selective 90° pulses. Pulses are applied with *x*-phase unless otherwise stated. The following delays are used: $\Delta = 1/(4J_{CC}) = 7.1$ ms, $T = 1/(2J_{CC}) = 14.1$ ms. The frequency selective pulses are applied with an EBURP[6] shape and with a length of 2 ms at 18.8 T. Deuterium, ^{2}H, decoupling is achieved with a continuous wave at a field of 860 Hz during the CEST period and at a field of 1.1 kHz during the rest of the experiment. A ^{13}C field strength between 20 and 60 Hz[13] is used during the 400 ms CEST period. IPAP[6] and post-processing are used for decoupling as described in Fig 2 and Supplementary Figure 2. Gradients of 1 ms are represented by black rectangles and applied with strength of g_1: 3.7 G/cm, g_2: 9.1 G/cm and g_3: 17.6 G/cm. Frequency discrimination is obtained by States-TPPI[3] of the phase ϕ_2. The phase cycle is ϕ_1: *x*, *x*, -*x*, -*x*; ϕ_2: *x*, -*x*; ϕ_{rec}: *x*, -*x*, -*x*, *x*. ***b*** Experimental CEST profile (red circles) along with least-squares fit (red line) of R125 ^{13}C$^{\delta}$ and T112 ^{13}C$^{\gamma 2}$ in T4L99A recorded at 278 K and 14.1 T. The following residue specific exchange parameters were obtained for R125: Ground state chemical shift, $\varpi_G = 43.622 \pm 0.014$ ppm, excited state chemical shift, $\varpi_E = 43.29 \pm 0.10$ ppm, $R_{1G} = R_{1E} = 0.322 \pm 0.013$ s^{-1}, $R_{2G} = R_{2E} = 7.6 \pm 0.6$ s^{-1}. For T112 the following parameters are obtained, $\varpi_G = 20.873 \pm 0.006$ ppm, $\varpi_E = 19.81 \pm 0.02$ ppm, $R_{1G} = R_{1E} = 0.171 \pm 0.011$ s^{-1}, $R_{2G} = 4.3 \pm 0.2$, and $R_{2E} = 5 \pm 20$ s^{-1}. Standard errors are obtained using the co-variance method[16] and additional details of analysis and least-squares fitting is given in the Material and Methods.

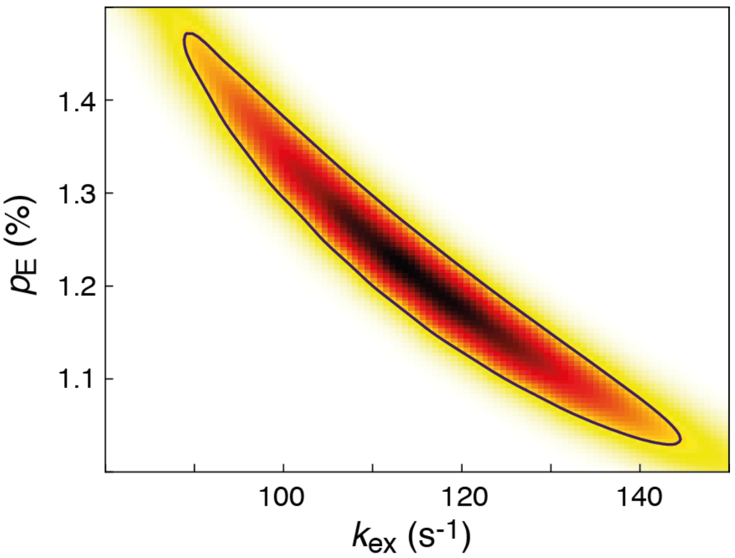

Supplementary Figure 10: Probability density surface for estimating of global exchange parameters of T4L99A at 278 K. The plot shows the probability surface from high to low (dark red to white) calculated as exp $(-(\chi^2 - \chi^2_{\min})/2)$. The CEST curves for the V103 and V111 were fit simultaneously for three CEST fields (ω_{CEST} = 19.8 Hz, 39.6 Hz, and 60.0 Hz) for fixed values of k_{ex} and p_E. The resulting χ^2 for each (k_{ex},p_E) point were used to generate the probability surface. The black line is drawn at a value where $\chi^2 - \chi^2_{\min} = 2.3$, which corresponds to one standard deviation in the two-parameter space.

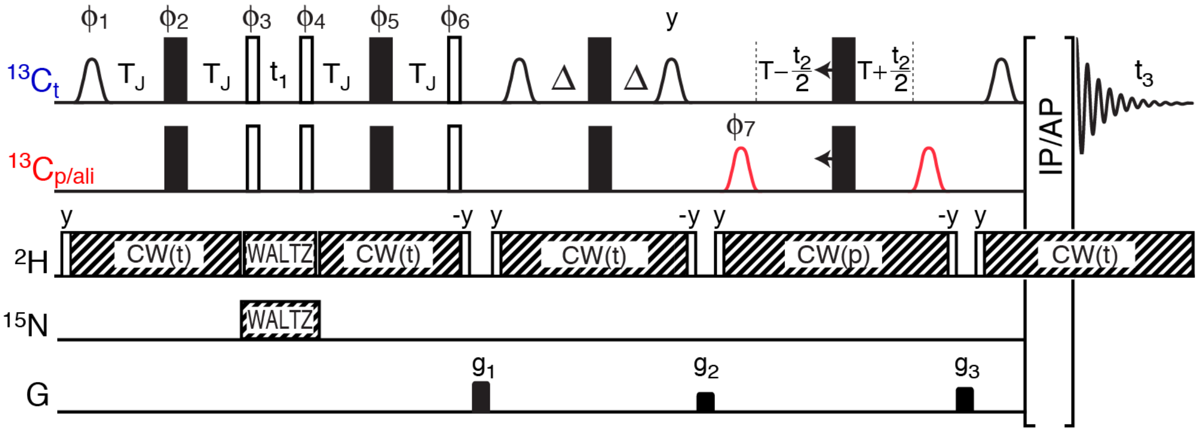

Supplementary Figure 11: Measurements of long-range $^3J_{CC}$ scalar couplings in side chains. The derived sequence follows the original idea by Bax and co-workers[14]. Black (open) bars represent 180° (90°) non-selective pulses, whereas open bell-shaped pulses represent frequency-selective 90° pulses. Red bell-shaped pulses are selective for the penultimate ^{13}C, that is, $^{13}C^{\gamma1}$ in isoleucine or $^{13}C^{\gamma}$ in arginine residues. The frequency selective 90° pulses are applied with an EBURP[2] shape with a length of 2 ms at 18.8 T. Deuterium, ^2H, decoupling is achieved with a continuous wave decoupling at a field of 1.1 kHz (CW(t) and CW(p)) or a WALTZ-16[11] decoupling at 1.1 kHz (WALTZ). The deuterium carrier is placed in the middle of the $^2H^{\delta}$ region (0.65 ppm for Ile, 3.1 ppm for Arg) during CW(t), in the middle of the $^2H^{\gamma}$ region (1.55 ppm for Ile and Arg) during CW(p) and in the middle of the aliphatic region (2.75 ppm) for WALTZ during the indirect chemical shift evolution t_1. See Supplementary Table 5 for a full list of carrier offsets. Flanking 90° pulses are placed before and after each ^2H decoupling block and the lock is not sampled during the first 1/3 of the recovery delay to achieve a stable lock signal[15]. The following delays are used: $\Delta = 1/(4J_{CC}) = 7.1$ ms, $T = 1/(2J_{CC}) = 14.1$ ms, $T_J = 1/J_{CC} = 28.2$ ms. Gradients of 1 ms are represented by black rectangles and applied with strength of g_1: 17.6 G/cm, g_2: 3.7 G/cm and g_3: 9.1 G/cm. The phase cycle is ϕ_1: *x*; ϕ_2: *x*, *y*; ϕ_3: 2(*y*), 2(-*y*); ϕ_4: 4(*y*),4(-*y*); ϕ_5: *x*; ϕ_6: *x*; ϕ_7: 4(*x*),4(-*x*); ϕ_{rec} = *x*, -*x*, *x*, -*x*, -*x*, *x*, -*x*, *x*. Frequency discrimination in t_1 is obtained by States-TPPI[3] of phases ϕ_1, ϕ_2, and ϕ_3, while the sign of ϕ_4, ϕ_5, and ϕ_6 is inverted every second t_1 time-increment along with an inversion of the receiver phase. Frequency discrimination in t_2 is obtained by States-TPPI[3] of phases ϕ_7. Examples of strips extracted from the three-dimensional experiment are shown in Fig. 5 and $^3J_{CC}$ derived for T4L L99A and MSG are shown in Supplementary Tables 3 and 4, respectively.

Supplementary Table 1: Longitudinal ^{13}C relaxation rates[a] of isoleucine $^{13}C^{\delta1}$ measured in T4L99A at 278 K

Nucleus	R_1 (s^{-1}) at 14.1 T	R_1 (s^{-1}) at 18.8 T
I3 $^{13}C^{\delta1}$	0.12 ± 0.02	0.15 ± 0.03
I9 $^{13}C^{\delta1}$	0.19 ± 0.02	0.23 ± 0.03
I17 $^{13}C^{\delta1}$	0.14 ± 0.02	0.09 ± 0.03
I27 $^{13}C^{\delta1}$	0.243 ± 0.012	0.253 ± 0.014
I29 $^{13}C^{\delta1}$	0.12 ± 0.02	0.15 ± 0.03
I50 $^{13}C^{\delta1}$	0.19 ± 0.02	0.06 ± 0.03
I58 $^{13}C^{\delta1}$	0.24 ± 0.02	0.22 ± 0.03
I78 $^{13}C^{\delta1}$	0.17 ± 0.03	0.168 ± 0.018
I100 $^{13}C^{\delta1}$	0.20 ± 0.02	0.12 ± 0.03
I150 $^{13}C^{\delta1}$	0.14 ± 0.06	0.13 ± 0.10

a) The non-selective longitudinal relaxation rates were measured using the pulse sequence shown in Supplementary Figure 5 with 11 relaxation delays {0.5 s, 7.0 s, 1.0 s, 6.0 s, 1.5 s, 5.0 s, 2.0 s, 4.0 s, 2.5 s, 3.5 s, 3.0 s} (14.1 T) or 10 relaxation delays {0.001 s, 8.0 s, 0.5 s, 6.0 s, 1.0 s, 4.0 s, 2.0 s, 3.0 s, 4.0 s, 1.0 s} (18.8 T). Standard derivations of the reported relaxation rates are obtained using the co-variance method[16].

Supplementary Table 2: Transverse ^{13}C relaxation rates[a] measured in T4L99A at 278 K

Nucleus	$R_{1\rho}$ (s^{-1})
**Arginine**	
R8 $^{13}C^{\delta}$	10.8 ± 0.4
R14 $^{13}C^{\delta}$	6.8 ± 0.2
R52 $^{13}C^{\delta}$	16.6 ± 1.1
R76 $^{13}C^{\delta}$	5.87 ± 0.15
R80 $^{13}C^{\delta}$	4.10 ± 0.11
R95 $^{13}C^{\delta}$	21.9 ± 1.9
R96 $^{13}C^{\delta}$	17.0 ± 0.9
R119 $^{13}C^{\delta}$	3.92 ± 0.08
R125 $^{13}C^{\delta}$	7.3 ± 0.3
R137 $^{13}C^{\delta}$	3.91 ± 0.10
R145 $^{13}C^{\delta}$	15.3 ± 1.2
R148 $^{13}C^{\delta}$	16.8 ± 1.2
R154 $^{13}C^{\delta}$	7.82 ± 0.15
**Isoleucine**	
I3 $^{13}C^{\delta1}$	2.9 ± 0.2
I9 $^{13}C^{\delta1}$	5.8 ± 0.4
I17 $^{13}C^{\delta1}$	6.6 ± 0.2
I27 $^{13}C^{\delta1}$	2.7 ± 0.4
I29 $^{13}C^{\delta1}$	3.6 ± 0.2
I50 $^{13}C^{\delta1}$	8.8 ± 1.6
I58 $^{13}C^{\delta1}$	4.9 ± 0.2
I78 $^{13}C^{\delta1}$	4.9 ± 0.2
I100 $^{13}C^{\delta1}$	4.02 ± 0.16
I150 $^{13}C^{\delta1}$	6.0 ± 0.6

a) The transverse relaxation rates were measured at 18.8 T in the rotating frame using the pulse sequence shown in Supplementary Figure 7, using a 1 kHz ^{13}C spin-lock field and 21 relaxation delays: {0.001 s, 0.104 s, 0.002 s, 0.096 s, 0.004 s, 0.088 s, 0.008 s, 0.080 s, 0.012 s, 0.072 s, 0.016 s, 0.064 s, 0.020 s, 0.056 s, 0.024 s, 0.048 s, 0.028 s, 0.040 s, 0.032 s, 0.008 s, 0.032 s, 0.096 s}. Standard derivations of the reported relaxation rates are obtained using the co-variance method[16].

Supplementary Table 3: Three-bond $^{13}C^{\delta}$-$^{13}C^{\alpha}$ scalar couplings measured for arginine residues in T4L99A

Residue	$^{3}J_{C\alpha C\delta}$ (Hz) [^{13}C detected]$^{a)}$ 298 K		278 K	$^{3}J_{C\alpha C\delta}$ (Hz) [^{1}H detected]$^{b)}$ 298 K
	**T = 14.1 ms**	**T = 28.1 ms**	**T=14.1 ms**	**T = 28.1 ms**
R8	4.66 ± 0.08	4.68 ± 0.05	4.78 ± 0.11	4.76 ± 0.13
R14	3.25 ± 0.05	3.23 ± 0.02	3.23 ± 0.08	3.10 ± 0.01
R52	5.84 ± 0.06	5.92 ± 0.09	5.53 ± 0.43	*N/A*
R76	5.15 ± 0.02	5.26 ± 0.02	5.33 ± 0.04	4.99 ± 0.03
R80	4.96 ± 0.02	5.02 ± 0.02	5.19 ± 0.04	4.89 ± 0.02
R95	5.60 ± 0.30	*N/A*	6.94 ± 0.81	*N/A*
R96	6.20 ± 0.15	*N/A*	6.86 ± 0.41	*N/A*
R119	4.68 ± 0.02	4.59 ± 0.02	4.74 ± 0.03	4.27 ± 0.06
R125	4.81 ± 0.04	4.90 ± 0.03	4.97 ± 0.08	4.74 ± 0.18
R137	4.41 ± 0.03	4.55 ± 0.02	4.80 ± 0.03	4.53 ± 0.02
R145	6.20 ± 0.08	6.16 ± 0.15	5.86 ± 0.40	*N/A*
R148	5.43 ± 0.08	5.90 ± 0.11	5.38 ± 0.41	*N/A*
R154	5.46 ± 0.02	5.51 ± 0.02	5.50 ± 0.05	5.49 ± 0.03

a) Obtained at 18.8 T on a uniformly isotope labelled [$^{2}H^{13}C^{15}N$]-T4L L99A sample using the pulse sequence in Supplementary Figure 11; experimental details in online methods. The standard deviations of the calculated $^{3}J_{CC}$ were estimated using a Monte-Carlo procedure to propagate the errors from the peak intensities, that in turn were estimated from the RMSD of a spectral region where no peaks were observed. b) Obtained at 18.8 T on a selectively isotope labelled [U-$^{2}H^{12}C$; Arg/Lys $^{1}H^{13}C^{15}N$]-T4L99A sample using a three-dimensional version of the method by Bax and co-workers[14]; experimental details in online methods. For the ^{1}H-detected experiments, the error was determined as the root-mean-square-deviation of two experiments.

Supplementary Table 4: Assignment and three-bond $^{13}C^{\delta1}$-$^{13}C^{\alpha}$ scalar couplings measured for isoleucine residues in MSG

Residue	$\delta(^{13}C^{\delta1})$ (ppm)[a)]	$\delta(^{13}C^{\gamma1})$ (ppm)[a)]	$^{3}J_{C\alpha C\delta1}$ (Hz)[b)]
I5	12.18	26.32	2.38±0.10
I12	14.81	26.92	3.3±0.3
I42	14.01	29.68	3.5±0.2
I60	11.49	29.75	3.6±0.2
I105	9.57	25.11	2.7±0.3
I109	13.84	27.67	3.3±0.3
I147	12.93	28.65	3.5±0.2
I148	10.66	25.86	2.1±0.3
I167	13.59	27.55	*N/A*
I200	15.46	28.47	<1.5[c]
I229	9.94	25.13	<1.5[c]
I238	13.05	26.88	2.8±0.2
I242	11.67	26.48	*N/A*
I248	11.62	28.01	3.8±0.5
I256	11.06	27.20	<2.5[c]
I260	10.51	27.34	<2.5[c]
I265	8.80	25.51	*N/A*
I268	12.91	26.70	3.0±0.4
I284	12.51	29.51	*N/A*
I309	12.62	26.12	2.6±0.4
I327	12.44	25.84	3.62±0.08
I337	13.93	28.47	2.0±0.3
I346	13.21	27.76	3.8±0.3
I349	14.23	28.70	3.0±0.5
I357	N/A	N/A	*N/A*
I361	12.09	27.37	*N/A*
I370	13.14	30.23	3.7±0.6
I388	11.96	26.28	*N/A*
I409	12.44	28.34	4.5 ± 0.7
I424	14.28	26.22	*N/A*
I439	13.05	29.05	3.4±0.5
I449	13.44	24.01	3.7±0.4

I482	13.76	29.78	4.0±0.3
I504	12.66	25.60	<2.1[c]
I560	13.11	27.17	3.5±0.4
I579	13.94	24.53	*N/A*
I592	13.06	28.37	3.8±0.4
I603	13.63	28.61	*N/A*
I615	11.97	25.63	3.26±0.13
I623	13.97	28.75	3.7±0.4
I637	14.01	28.02	*N/A*[d]
I642	15.27	26.77	<2.5[c]
I650	7.48	26.45	<2.1
I697	14.03	28.05	*N/A*[d]

a) Assignment based on the assignment by Tugarinov and Kay[8,17] and the 3D-CC TOCSY experiment, Fig. 2b and Supplementary Figure 8, and correlations in the 3D $^{3}J_{C\alpha C\delta 1}$ experiment. b) The standard deviations of the calculated $^{3}J_{CC}$ were estimated using a Monte-Carlo procedure to propagate the errors from the peak intensities, that in turn were estimated from the RMSD of a spectral region where no peaks were observed. c) Only diagonal peak, $^{13}C^{\delta 1}$-$^{13}C^{\gamma 1}$-$^{13}C^{\delta 1}$ observed and a maximum value for $^{3}J_{C\alpha C\delta 1}$ has been estimated from the intensity of the diagonal peak and the noise level. d) I637 and I697 are overlapped in the $^{13}C^{\delta 1}$-$^{13}C^{\gamma 1}$ spectrum.

Supplementary Table 5: Carrier frequencies used for obtaining residue-specific ^{13}C-^{13}C correlation spectra[a].

Experiment	$^{13}C_t$ (ppm)	$^{13}C_p$ (ppm)	$^{2}H_t$ (ppm)[b]	$^{2}H_p$ (ppm)[b]	$^{1}J_{CC}$ (Hz)[c]
Arg 2D $^{13}C^{\delta}$-$^{13}C^{\gamma}$<br>Lys 2D $^{13}C^{\varepsilon}$-$^{13}C^{\delta}$	42.65	26.55	3.11	1.55	35.8 ± 0.3<br>36.4 ± 1.0
Ile 2D $^{13}C^{\delta 1}$-$^{13}C^{\gamma 1}$	12.65	26.55	0.75	1.00	35.2 ± 0.5
Ile 2D $^{13}C^{\gamma 2}$-$^{13}C^{\beta}$	16.65	38.05	0.78	1.78	36.1 ± 0.5
Pro 2D $^{13}C^{\delta}$-$^{13}C^{\gamma}$	52.15	28.65	3.64	1.91	34.2 ± 0.4
Thr 2D $^{13}C^{\gamma 2}$-$^{13}C^{\beta}$	19.40	68.90	1.14	4.16	36.4 ± 0.5
Val 2D $^{13}C^{\gamma 1,2}$-$^{13}C^{\beta}$	20.65	31.65	0.65	1.83	35.6 ± 1.1

a) All chemical shifts are referred to DSS (4,4-dimethyl-4-silapentane-1-sulfonic acid). For referencing to TMS (tetramethylsilane), which is often used by Bruker spectrometers, 2.65 ppm[18] is subtracted from all ^{13}C chemical shifts listed. b) The ^{2}H carrier frequencies are obtained from the BMRB[1]. c) The one-bond couplings were measured in 2D ^{13}C-^{13}C correlation spectra as the splitting between the two components of the doublet. Average and standard deviation of the observed couplings are given.

Supplementary References:

1. Ulrich, E. L. *et al.* BioMagResBank. *Nucleic Acids Res.* **36,** D402–D408 (2008).
2. Geen, H. & Freeman, R. Band-selective radiofrequency pulses. *J. Magn. Reson.* **93,** 93–141 (1991).
3. Marion, D., Ikura, M., Tschudin, R. & Bax, A. Rapid recording of 2D NMR spectra without phase cycling. Application to the study of hydrogen exchange in proteins. *J. Magn. Reson.* **85,** 393–399 (1989).
4. Bouvignies, G. *et al.* Solution structure of a minor and transiently formed state of a T4 lysozyme mutant. *Nature* **477,** 111–117 (2011).
5. Yamazaki, T., Muhandiram, R. & Kay, L. E. NMR experiments for the measurement of carbon relaxation properties in highly enriched, uniformly ^{13}C, ^{15}N labeled proteins: application to $^{13}C^{a}$ carbons. *J. Am. Chem. Soc.* **116,** 8266–8278 (1994).
6. Bermel, W., Bertini, I., Felli, I. C., Kümmerle, R. & Pierattelli, R. Novel ^{13}C direct detection experiments, including extension to the third dimension, to perform the complete assignment of proteins. *J. Magn. Reson.* **178,** 56–64 (2006).
7. Tugarinov, V. & Kay, L. E. Ile, Leu, and Val methyl assignments of the 723-residue malate synthase G using a new labeling strategy and novel NMR methods. *J. Am. Chem. Soc.* **125,** 13868–78 (2003).
8. Tugarinov, V., Muhandiram, R., Ayed, A. & Kay, L. E. Four-dimensional NMR spectroscopy of a 723-residue protein: Chemical shift assignments and secondary structure of malate synthase G. *J. Am. Chem. Soc.* **124,** 10025–10035 (2002).
9. McCoy, M. A. & Mueller, L. Selective Shaped Pulse Decoupling in NMR - Homonuclear ^{13}C carbonyl decoupling. *J. Am. Chem. Soc.* **114,** 2108–2112 (1992).
10. Mohebbi, A. & Shaka, A. J. Improvements in C-13 Broad-band Homonuclear Cross-Polarization for 2D and 3D NMR. *J. Chem. Phys.* **178,** 374–377 (1991).
11. Shaka, A. J., Keeler, J., Frenkiel, T. & Freeman, R. An improved sequence for broadband decoupling: WALTZ-16. *J. Magn. Reson.* **52,** 335–338 (1983).
12. Vallurupalli, P., Bouvignies, G. & Kay, L. E. Studying ‘invisible’ excited protein states in slow exchange with a major state conformation. *J. Am. Chem. Soc.* **134,** 8148–61 (2012).
13. Bouvignies, G., Vallurupalli, P. & Kay, L. E. Visualizing Side Chains of Invisible Protein Conformers by Solution NMR. *J. Mol. Biol.* **426,** 763–774 (2014).
14. Bax, A., Max, D. & Zax, D. Measurement of long-range ^{13}C-^{13}C J couplings in a 20

kDa protein-peptide complex. *J. Am. Chem. Soc.* **114,** 6923–6925 (1992).

15. Gardner, K. H., Konrat, R., Rosen, M. K. & Kay, L. E. A (H)C(CO)NH-TOCSY pulse scheme for sequential assignment of protonated methyl groups in otherwise deuterated ^{15}N,^{13}C labeled proteins. *J. Biomol. Nmr* **8,** 351–356 (1996).
16. Press, W. H., Teukolsky, S. A., Vetterling, W. T. & Flannery, B. P. *Numerical Recipes in C. Technometrics* **29,** (1992).
17. Tugarinov, V. & Kay, L. E. Quantitative NMR studies of high molecular weight proteins: application to domain orientation and ligand binding in the 723 residue enzyme malate synthase G. *J. Mol. Biol.* **327,** 1121–1133 (2003).
18. Zhang Shanmin. Auto-offsetting and -referencing in biomolecular NMR experiments. *J. Biophys. Chem.* **2,** 208–214 (2011).
