# Supplementary material for: Characterising side chains in large proteins by protonless 13C-detected NMR spectroscopy
Source: Nat Commun. 2019 Apr 15;10:1747. doi: 10.1038/s41467-019-09743-4 (PMC6465260; doi:10.1038/s41467-019-09743-4)
Supplement: Supplementary file 3 — Reporting Summary [file 41467_2019_9743_MOESM3_ESM.pdf]

nature research

Corresponding author(s): Prof. D. Flemming Hansen

Last updated by author(s): Mar 22, 2019

Reporting Summary

Nature Research wishes to improve the reproducibility of the work that we publish. This form provides structure for consistency and transparency in reporting. For further information on Nature Research policies, see Authors & Referees and the Editorial Policy Checklist.

Statistics

For all statistical analyses, confirm that the following items are present in the figure legend, table legend, main text, or Methods section.

n/a	Confirmed	
☐	☒	The exact sample size (*n*) for each experimental group/condition, given as a discrete number and unit of measurement
☐	☒	A statement on whether measurements were taken from distinct samples or whether the same sample was measured repeatedly
☒	☐	The statistical test(s) used AND whether they are one- or two-sided<br>*Only common tests should be described solely by name; describe more complex techniques in the Methods section.*
☐	☒	A description of all covariates tested
☒	☐	A description of any assumptions or corrections, such as tests of normality and adjustment for multiple comparisons
☐	☒	A full description of the statistical parameters including central tendency (e.g. means) or other basic estimates (e.g. regression coefficient) AND variation (e.g. standard deviation) or associated estimates of uncertainty (e.g. confidence intervals)
☒	☐	For null hypothesis testing, the test statistic (e.g. *F*, *t*, *r*) with confidence intervals, effect sizes, degrees of freedom and *P* value noted<br>*Give P values as exact values whenever suitable.*
☒	☐	For Bayesian analysis, information on the choice of priors and Markov chain Monte Carlo settings
☒	☐	For hierarchical and complex designs, identification of the appropriate level for tests and full reporting of outcomes
☒	☐	Estimates of effect sizes (e.g. Cohen's *d*, Pearson's *r*), indicating how they were calculated

Our web collection on statistics for biologists contains articles on many of the points above.

Software and code

Policy information about availability of computer code

Data collection	Bruker Avance III HD and Avance II NMR spectrometer, Topspin 3.2, Topspin 3.5
Data analysis	nmrPipe version 9.6, NMRFAM-Sparky version 1.413, CCPM version 3, numpy (python 2.7.9) version 1.15.2, gnuplot version 5.2 pl 2

For manuscripts utilizing custom algorithms or software that are central to the research but not yet described in published literature, software must be made available to editors/reviewers. We strongly encourage code deposition in a community repository (e.g. GitHub). See the Nature Research guidelines for submitting code & software for further information.

Data

Policy information about availability of data

All manuscripts must include a data availability statement. This statement should provide the following information, where applicable:

- Accession codes, unique identifiers, or web links for publicly available datasets
- A list of figures that have associated raw data
- A description of any restrictions on data availability

All pulse programs (sequences) are available from the corresponding author upon request

Field-specific reporting

Please select the one below that is the best fit for your research. If you are not sure, read the appropriate sections before making your selection.

☒ Life sciences ☐ Behavioural & social sciences ☐ Ecological, evolutionary & environmental sciences

For a reference copy of the document with all sections, see nature.com/documents/nr-reporting-summary-flat.pdf

Life sciences study design

All studies must disclose on these points even when the disclosure is negative.

Sample size	Number of points acquired in the NMR spectra are detailed in the methods section. The number of points sampled in the NMR spectra was decided from standard considerations e.g. measurement time and signal-to-noise. A detailed explanation is given in Fig S3 and S4 were two different sample sizes are compared (For the two-dimensional carbon-carbon correlation spectra)
Data exclusions	As detailed in supplementary information, for the scalar coupling experiments, when the peak intensity is within the noise the corresponding data point was not included. Otherwise no data were excluded.
Replication	Multiple measurements were used to calculate the uncertainties for both spin-relaxation and scalar coupling measurements. Scalar couplings for T4 lysozyme were also measured at two different magnetic field strengths (two different spectrometers) to further verify data.
Randomization	Not used
Blinding	Not used

Reporting for specific materials, systems and methods

We require information from authors about some types of materials, experimental systems and methods used in many studies. Here, indicate whether each material, system or method listed is relevant to your study. If you are not sure if a list item applies to your research, read the appropriate section before selecting a response.

Materials & experimental systems

n/a	Involved in the study
☒	☐ Antibodies
☒	☐ Eukaryotic cell lines
☒	☐ Palaeontology
☒	☐ Animals and other organisms
☒	☐ Human research participants
☒	☐ Clinical data

Methods

n/a	Involved in the study
☒	☐ ChIP-seq
☒	☐ Flow cytometry
☒	☐ MRI-based neuroimaging
