# Supplementary material for: Characterising side chains in large proteins by protonless 13C-detected NMR spectroscopy
Source: Nat Commun. 2019 Apr 15;10:1747. doi: 10.1038/s41467-019-09743-4 (PMC6465260; doi:10.1038/s41467-019-09743-4)
Supplement: Supplementary file 2 — Peer Review [file 41467_2019_9743_MOESM2_ESM.pdf]

Reviewers' comments:

Reviewer #1 (Remarks to the Author):

The manuscript demonstrates the utility of direct 13C NMR detection to characterize structure and dynamics of substantial-size proteins. As the authors point out, so far the use of low-gamma detected NMR technology has been largely restricted to IDPs. Here, they convincingly demonstrate utility of this approach for two systems where conventional 1H-detected experiments would be challenging or impossible. Their work demonstrates that with clever pulse sequence design, one can take advantage of the better 2D dispersion of 13C-13C correlation maps to reduce spectral overlap, whereas the improved slower transverse relaxation more than offsets the anticipated, disadvantageous lengthening of T1. Their demonstration that one can get detailed chi2 information on Arg or Ile residues, and measure slow exchange processes by 13C CEST opens effectively a new level of structural biology: For the first time, we can start studying details of intermolecular contacts, including the dynamic distribution of sidechains under physiological conditions, at room temperature. This work is novel, and likely will become a landmark in NMR studies of proteins and I therefore enthusiastically support its publication after a few minor issues are addressed.

1. P.2, line 42-43: it's not immediately clear what the authors mean to convey with (i-ii) etc. Even if the reader is lucky enough to find these markings in Fig1, he/she is unlikely to figure out what is intended.

2. The S/N impact of the IP/AP artificial decoupling relative to conventional decoupling has been described in the literature, but depending on where one looks, the conclusions are different. It might be useful to point out that root_2 improvement in S/N is obtained relative to no decoupling (and root2 is lost relative to conventional decoupling).

3. The authors emphasize in numerous places how rapidly they can record their T4L spectra, but don't list the sample concentration. This should be included. It is included for MSG, but there the measurement times are longer and S/N is lower.

4. P.4, legend line 64/65. It seems that obtaining the chemical shift assignment still requires the earlier 1H-detected backbone experiments. Pointing this out more explicitly may be helpful.
5. P4. Line 71/72: "all the" seems not quite correct: the left panels were acquired in 12 minutes, the right panels in 38 minutes.

6. P4, line 77: "substantialLY better chemical shift dispersion", This statement really applies to 13C-13C correlation maps (compared to 1H-13C). The chemical shift dispersion of 13C itself is of course unchanged.

7. Table S3: the 3JCC couplings for many residues appear unrealistically large (>6Hz). The authors should verify whether they were calculated correctly. I suspect a factor of 0.5 (or 2) to be missing somewhere.

8. Fig S3: "minimal sweep width" Please add between brackets what value was actually used.
9. Although it's obvious to readers who understand the pulse schemes, perhaps not so obvious to those less experienced: Mark where the time starts/ends (e.g T-t2/2; add some vertical dashed lines?). For Fig S9, t1 is part of a constant-time evolution, but this is not indicated in the sequence. FLOPSY really applies to all aliphatic carbons, not just Ct. Explicitly spelling this out somewhere (caption?) may be helpful.

Reviewer #2 (Remarks to the Author):

The authors present a suite of related NMR pulse sequences that each enables monitoring a defined protein side-chains at the atomic level in larger proteins. Thus, the many applications of NMR in studies of large proteins now becomes accessible to a much larger number of chemical moieties, such as delta, gamma, or epsilon carbons. This is a considerable advance. Of note, the methods presented are spectroscopically straightforward (the authors rely on selective INEPT transfers for the "root" experiment and expand them with established experiments), but this work does provide new important tools and the authors nicely demonstrate feasibility. Significance is strengthened by introducing novel experiments to monitor side-chain dynamics and conformations. I believe the manuscript will be of interest to a substantial fraction of Nat. Comm.'s readership with minor modifications to either use descriptions more in line with this targeted readership or to allow for ease of reproducibility. I tried to separate my comments according the these two objectives.

(i) Certain aspects may be difficult to follow by the general readership of Nat. Comm. and may impede the impact of this otherwise important manuscript. They can be clarified by simple rewording.

The authors should clearly state that they offer a series of experiments that monitor side-chains for a subset of residue types and not all residue types. This should already be apparent in the abstract and detailed in the main text. Notably, please list all side-chain moieties and types that are now accessible. The latter can be resolved by replacing the "etc" on page 2, line 41 with an exhaustive list. See also the related comment in (ii) below.

I think the authors' success relies on starting with and ending magnetization transfers on terminal carbon moieties as this strategy bypasses evolution under multiple carbon-carbon couplings that would otherwise deteriorate the quality of the spectra, both because of sensitivity losses and crowding. This is an important aspect and the authors should explicitly describe this advantage.

In Figure 1a, shouldn't the bars for alpha and beta be gray, for consistency with the SI?

When possible the authors should describe or comment on the biological functional significance of their findings regarding the various residues they pinpoint through CEST, 3JCC measurements, etc. What is interesting about your observations for V103, R14, R148?

A NatComm reader will need more hand-holding during the description of 3Jcc (pages 5 and 6). Briefly reminding the reader of Karplus and briefly explaining how dynamics are assessed through 3Jcc should do the job. Or is the reference to dynamics because 3Jcc are needed to fit CEST? Both are true.

What did you learn about MSG? The text about 3Jcc ends with a vague statement.

T1 and T1rho experiments are hidden in the SI and not really discussed. Were they designed (and required) to get R1 and R2 values for CEST analysis? When reading the main text, the reader should at least learn that they exist.

(ii) The following must be resolved, clarified, or simplified to facilitate reproducibility.

For clarity, and to make sure that no parameter has been left out, I would duplicate some information from the Method section in the supporting information, as relevant, so that a complete description can be found in one place, in the SI. Notably, information for the "root" pulse sequence is spread between Figure 1, the Method, and SI Figure S2. You should consolidate all information needed for

reproducibility in the caption of S2, including a (very brief) description of the IPAP block.

Rather than giving examples, please provide a table for all 13C and 2H carrier frequencies for all residue-specific moieties. I imagine some effort went into determining these numbers and the quality of the correlation maps will depend on them.

Please specify that chemical shifts (and carrier frequencies) are referenced with respect to DSS (if they are). This information is particularly relevant for implementing the pulse sequence as some Bruker installations come with TMS when others come with DSS as default (irrespective of subsequent corrections during analysis).

Please specify that the 13C carrier is shifted according to 90 shaped pulses on Ct and Cp in all cases unless otherwise indicated (if true).

Line 225: include "TCI" (I believe the probe is a TCI?)

When describing the encoding of indirect dimensions, the term "real points" would imply that as many imaginary points were collected. If so, please state explicitly. If not, replace with "complex points".

In S5, the authors measure recovery from transverse magnetization rather than inversion recovery. A little note and reference is needed here.

In all relaxation experiments, provide the exact values of relaxation times (mixing times) and the order in which they were acquired (including if interleaved mixing time/quadrature acquisition was used).

Typos:
Line 255: giving -> given.

SI: Figure S1: swap Cd-Ce with Ce-Cd (for consistency with your notation elsewhere).

S9: "during rest of" -> "during the rest of"

Reviewer #3 (Remarks to the Author):

The paper by Pritchard and Hansen proposes 13C detected NMR experiments to study protein side chains. The proposed 2D NMR experiment correlates 13C chemical shift of terminal and penultimate carbons of amino acid side chains. The observation made by authors that the two chemical shifts together are a unique signature for an amino acid, and provides an opportunity to simplify spectrum by focussing on one amino acid. They have listed Ile, Val, Thr, Pro, Arg, and Lys having such characteristic chemical shifts. The data has been presented for all amino acids except Lys. Also, there can be some contamination in the spectrum of Ile from Val and in Arg from Lys. Such correlation maps require to prepare only a single sample. This base experiment is extended to design experiments to included CEST and three bond scalar couplings, which provide information on the structure and dynamics of side chains. Yet another solid price of work from the Hansen group. However, I am unable to see the novelty in this work that warrants Nature Communications. There are other NMR methods available to extract the same data.

Some major point to be addressed by the authors.

a) As discussed in the paper, the pulse sequence starts with magnetization from 13C and detects magnetization on 13C. This makes the pulse sequence very insensitive. However, they claimed to

measure experiment within 12 mins by applying spectral folding/aliasing. I think they should report this along with the sample concentrations to prevent any confusion.

b) They should also discuss the effect of homonuclear coupling in the measurement of T1p and CEST. There is a paper by Kay et.al which talks about in general negligible effect of 13C 13C homonuclear coupling in CEST measurements. Are there any studies for T1p?

c) The decoupling sought by IPAP depends on the 13C-13C scalar coupling. Is it safe to assume 35 Hz for all side chains 13C-13C coupling? If this is not the case it can distort the line shapes.

d) The recommended interscan delay is 3T1 to 5T1 which amount to 30 to 50 seconds in these experiments. However, they have recorded with an interscan delay of 4 and 10 seconds. This would have impacted the sensitivity. They may improve sensitivity per unit time by flipping magnetization for less than 90 degrees.

e) Finally, authors can write a few lines on what extra information such correlations can provide which cannot be got from by existing experiments which study 1H, 13C correlation of terminal methyls. Of course, one thing is that the indirect dimension has better chemical shift dispersion by using 13C instead of 1H.

Minor Comments:

1. On page 4 "..characterize many side chains." The number is 6, and 5 without interference from other amino acids.

2. Fig S5 for measuring T1, the first pulse is 90 degrees. In the inversion recovery, the magnetization is flipped to 180 degrees.

3. Fig s4 (b), two peaks are assigned 145. One of them should be 148.

4. Please mention the units in Table S3.

5. On page 11, "..experimental details are giving in the legend"
should be "..are given"

6. The caption of Fig S12, "fellows the original idea" should be "follows"

7. The work of Dominique Marion on out and back TOCSY should be mentioned. J Biomol NMR. 2015 Dec;63(4):389-402

Reviewer 1:

1. P.2, line 42-43: it's not immediately clear what the authors mean to convey with (i-ii) etc. Even if the reader is lucky enough to find these markings in Fig1, he/she is unlikely to figure out what is intended.

We agree that the statement could be challenging to follow for non-specialists. The figure that the reviewer is referring to is now Figure 2. We have changed the sentence to:

"In the core experiment, Fig. 2a and Supplementary Figure 2, magnetisation is initially transferred from $^{13}C_t$ *to* $^{13}C_p$*, then labelled with the chemical shift of* $^{13}C_p$ *during* t_1 *and finally transferred back to* $^{13}C_t$ *for detection."*

Figure 2 and the legend have also been modified to make this point more clear.

"The flow of the magnetisation between $^{13}C_t$ *(blue) and* $^{13}C_p$ *(red) is shown above the sequence with colour gradients."*

2. The S/N impact of the IP/AP artificial decoupling relative to conventional decoupling has been described in the literature, but depending on where one looks, the conclusions are different. It might be useful to point out that root_2 improvement in S/N is obtained relative to no decoupling (and root2 is lost relative to conventional decoupling).

We have now included a brief description of the IPAP block in Supplementary Figure 2 and specifically added a comment about signal-to-noise:

"The IPAP block is shown below the pulse sequence, where the two sub-spectra required for IPAP were acquired before the t_1 *loop. Virtual decoupling using IPAP leads to half the number of peaks in the spectrum and an improvement in signal-to-noise by a factor of* $\sqrt{2}$ *compared to no decoupling."*

3. The authors emphasize in numerous places how rapidly they can record their T4L spectra, but don't list the sample concentration. This should be included. It is included for MSG, but there the measurement times are longer and S/N is lower.

We completely agree with this reviewer and with reviewer 3 that the concentration of the T4L L99A sample used here should be included in the main manuscript. We have now added this to the legend of Figure 2,

"… obtained on a 1.4 mM sample .."

In the method section we have added:

"The NMR sample contained 1.4 mM protein in 95%/5% $^{1}H_2O$ / $^{2}H_2O$ *for the uniformly* [$^{2}H^{13}C^{15}N$] *labelled sample and 1.5 mM protein in 100%* $^{2}H_2O$ *for the* [$^{1}H^{13}C$*-Lys,Arg;* $^{2}H^{12}C$] *labelled sample."*

In the legend to Supplementary Figure 3 and 4:

" ... using a 1.4 mM sample of T4L L99A .. "

4. P.4, legend line 64/65. It seems that obtaining the chemical shift assignment still requires the earlier 1H-detected backbone experiments. Pointing this out more explicitly may be helpful.

We have now added in the method section that the previous chemical shift assignment of MSG - used to assign the ^{13}C-^{13}C spectra - was obtained from ^{1}H-detected backbone experiments.

"... was transferred from a previously published assignment[26,27], that in turn was based on ^{1}H-detected experiments."

5. P4. Line 71/72: "all the" seems not quite correct: the left panels were acquired in 12 minutes, the right panels in 38 minutes.

We have removed 'all', and also specified the number of spectra presented:

" (2) six different ^{13}C-^{13}C correlation maps can be obtained in less than 12 min (see Supplementary Figures 3 and 4),"

6. P4, line 77: "substantialLY better chemical shift dispersion", This statement really applies to 13C-13C correlation maps (compared to 1H-13C). The chemical shift dispersion of 13C itself is of course unchanged.

We have now changed the sentence to make this comparison explicitly between ^{13}C-^{13}C spectra and ^{1}H-^{13}C spectra:

"Another striking advantage of ^{13}C-^{13}C correlation spectra compared to ^{1}H-^{13}C spectra is the substantially better chemical shift dispersion in the directly detected dimension resulting in significantly better peak separation, "

7. Table S3: the 3JCC couplings for many residues appear unrealistically large (>6Hz). The authors should verify whether they were calculated correctly. I suspect a factor of 0.5 (or 2) to be missing somewhere.

These large $^{3}J_{CC}$ couplings observed in arginine residues are substantially larger than those observed for methyl-bearing side chains. Based theoretical density functional theory (DFT) calculations and ^{13}C spectra of free L-arginine in solution we are convinced that the numbers are correct and that we are most-likely not missing a factor of 2.

(a) [Unpublished]: DFT calculations on an N-acetylated and C-NHCH3 capped fragment of L-arginine show a $^3J_{CaCd}$ coupling of 5.64 Hz, when the χ_2 dihedral angle is 180° (*trans*). These calculations were carried out using the B3LYP functional and a EPR-III basis set in the Gaussian16 programme.

(b) [Unpublished]: High-resolution one-dimensional ^{13}C spectra of [U-^{1}H^{13}C^{15}N] L-arginine shows a 5.03 Hz long-range scalar coupling from $^{13}C^{\delta}$ and a 5.08 Hz coupling from $^{13}C^{\alpha}$, which we assign to the $^3J_{CaCd}$. (see below). This agrees with the fact that the χ_2 angle of arginine residues in a random coil state is expected to sample the *trans* conformation about 80% (Lovell *et al.*, PMID: 10861930) of the time and so the scalar coupling observed for a full *trans* conformation is expected to be ≳ 6 Hz.

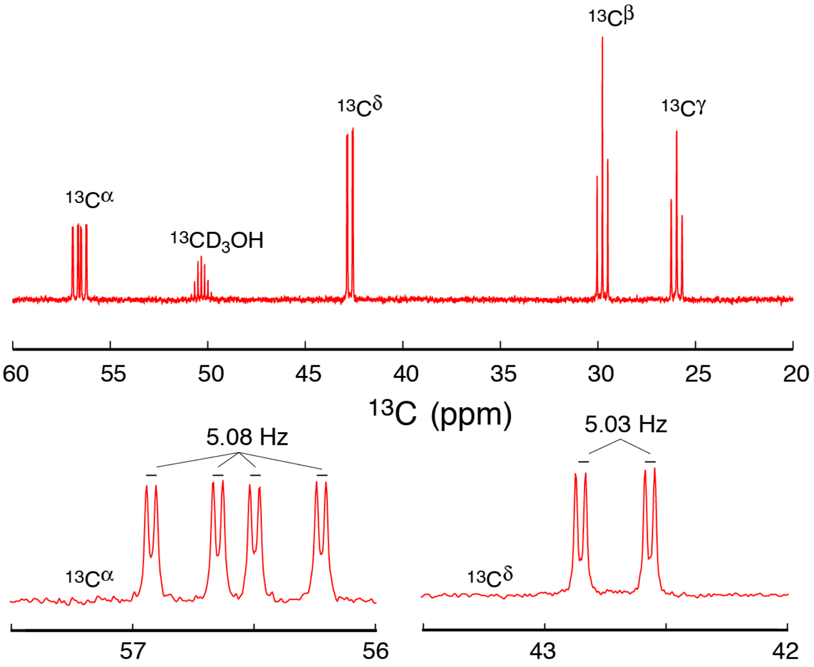

Figure: One-dimensional ^{13}C NMR spectrum of $^{13}C_6{}^{15}N_4$ L-arginine, recorded at 298 K, 125.8 MHz (500 MHz ^{1}H spectrometer). An acquisition time of 450 millisecond was used and ^{15}N and ^{1}H decoupling was applied during the acquisition. The spectrum shows long-range $^3J_{CC}$ between $^{13}C^{\delta}$ and $^{13}C^{\alpha}$. Spectrum is referenced to DSS.

Although the DFT calculations and NMR spectrum above clearly justify that the long-range $^3J_{CC}$ can take the values given in Supplementary Table 3, we do not think these data will improve the manuscript for the general readership of Nature Comm.

A separate manuscript is in preparation, where the scalar couplings of arginine side chains is discussed in details, with the aim to derive information about structure and dynamics.

8. Fig S3: "minimal sweep width" Please add between brackets what value was actually used.

This has now been added to both Supplementary Figure 3 and 4.

In Supplementary Figure 3:

*"… (4 ppm for **a**,**b**,**d** and 3 ppm for **c**), …"*

In Supplementary Figure 4:

" … (4 ppm) … "

9. Although it's obvious to readers who understand the pulse schemes, perhaps not so obvious to those less experienced: Mark where the time starts/ends (e.g T-t2/2; add some vertical dashed lines?).

We have now added vertical dashed lines to all the sequences in Supplementary Material, Supplementary Figures 2, 5, 7, 8, 9, and 11

For Fig S9, t1 is part of a constant-time evolution, but this is not indicated in the sequence.

In Supplementary Figure 8 (previous S9), the chemical shift evolution during t_1 is not constant time – the chemical shift evolution during t_2 is constant time. We can see that the sequence could look as if constant time was used and the following has therefore been added to the legend of Supplementary Figure 8:

"The second ^{13}CO selective pulse during t_1 provides a Bloch–Siegert compensation for the first ^{13}CO pulse."

"Chemical shift evolution during t_1 is non-constant time, while chemical shift evolution during t_2 is recorded in a constant time manner."

FLOPSY really applies to all aliphatic carbons, not just Ct. Explicitly spelling this out somewhere (caption?) may be helpful.

It was already noted in the caption to Supplementary Figure 8 that the FLOPSY element was applied to all aliphatic ^{13}C. "The ^{13}C carrier is placed at 42 ppm during t_1 and isotropic FLOPSY mixing and moved to $^{13}C_t$ and $^{13}C_p$ during rest of sequence.". To make this more clear, we have now also modified the figure and added the FLOPSY block to the line for $^{13}C_p$ and added:

" … with a FLOPSY-8 scheme applied at a field strength of 7.8 kHz for 18 ms and centered at 42 ppm"

Reviewer 2:

(i) Certain aspects may be difficult to follow by the general readership of Nat. Comm. and may impede the impact of this otherwise important manuscript. They can be clarified by simple rewording.

Please see below how we have adjusted the manuscript according the each of the reviewers points in order to make to manuscript easier to follow by the general readership of Nature Communications.

The authors should clearly state that they offer a series of experiments that monitor side-chains for a subset of residue types and not all residue types. This should already be apparent in the abstract and detailed in the main text. Notably, please list all side-chain moieties and types that are now accessible. The latter can be resolved by replacing the "etc" on page 2, line 41 with an exhaustive list. See also the related comment in (ii) below.

We have now extended the abstract and also included that in this work we characterize six different types of amino acid side chains:

" *... A single, uniformly isotopically labelled sample is sufficient to characterise the side chains of six different amino acid types. Side-chain conformational dynamics on the millisecond time-scale can be quantified by incorporating chemical exchange saturation transfer (CEST) into the presented methods, whilst long-range* ^{13}C-^{13}C *scalar couplings reporting on the nanosecond to millisecond motions of side chains can be quantified in proteins with sizes up to 80 kDa. The presented class ...*"

We have also included a complete list of the side-chain moieties that can currently be characterised on page 2:

"*The terminal* $^{13}C_t$ *include:* $^{13}C^{\delta}$ *in the arginine side chain,* $^{13}C^{\delta 1}$ *and* $^{13}C^{\gamma 2}$ *in isoleucine,* $^{13}C^{\varepsilon}$ *in lysine,* $^{13}C^{\delta}$ *in proline,* $^{13}C^{\gamma 2}$ *in threonine, and* $^{13}C^{\gamma}$ *in the valine residues.* "

I think the authors' success relies on starting with and ending magnetization transfers on terminal carbon moieties as this strategy bypasses evolution under multiple carbon-carbon couplings that would otherwise deteriorate the quality of the spectra, both because of sensitivity losses and crowding. This is an important aspect and the authors should explicitly describe this advantage.

The reviewer is absolutely correct. The success of the new method hinge currently on the fact that we start with a 'terminal' carbon and also detect on the same terminal carbon. The transfer of magnetisation from $^{13}C_t$ to $^{13}C_p$ can then be carried out using a simple INEPT, and a **single** IPAP element can be used for virtual decoupling in the direct dimension. We have now stressed this further in the manuscript.

"The fact that the magnetisation of interest starts and is detected on the terminal $^{13}C_t$ *has some key advantages. The homonuclear coupling pattern for* $^{13}C_t$ *is a simple doublet, which allows for the magnetisation of interest to be transferred completely*

between $^{13}C_t$ and $^{13}C_p$ using simple INEPTs and also facilitates virtual decoupling in the $^{13}C_t$ detected dimension.."

In Figure 1a, shouldn't the bars for alpha and beta be gray, for consistency with the SI?

We have now changed Figure 1 as suggested by the reviewer.

When possible the authors should describe or comment on the biological functional significance of their findings regarding the various residues they pinpoint through CEST, 3JCC measurements, etc. What is interesting about your observations for V103, R14, R148?

In the original manuscript, the CEST experiments on V103 of T4L L99A were discussed in terms of the structural information that can be obtained for the excited state. We have now also added a comment about the dynamics and the distribution of rotameric states that becomes available with the new experiment.

"Access to the ^{13}C chemical shifts in the excited state via the ^{13}C-detected CEST experiment also reveals that the V103 side chain is more dynamic in the excited state with a broader distribution of side-chain rotamers.."

For the $^3J_{CC}$ measurements we have added the following for R14 and R148 of T4L L99A:

"For the two arginine side chains of T4L L99A shown in Fig 5a, the intermediate value of the coupling constant for R14 shows that this side chain is flexible and dynamic around the χ_2 angle, while the high value observed for the R148 side chain shows a rigid trans conformation. This is in good agreement with previous characterisations of the dynamics of the arginine side chains of T4L L99A[6,31,32] as well as with the structure of T4L L99A, where R14 is on the surface and R148 is engaged in a salt-bridge with D10."

A NatComm reader will need more hand-holding during the description of 3Jcc (pages 5 and 6). Briefly reminding the reader of Karplus and briefly explaining how dynamics are assessed through 3Jcc should do the job. Or is the reference to dynamics because 3Jcc are needed to fit CEST? Both are true.

We have now added the following sentence:

"The three-bond scalar coupling $^3J_{C\alpha C\delta}$ relates to the side chain χ_2 dihedral angle via a Karplus relationship, where large coupling constants are observed when the χ_2 angle is in a trans conformation and small couplings are observed when χ_2 is in a gauche conformation. Intermediate values of the coupling constant are observed when the side chain is dynamic around the χ_2 dihedral angle."

What did you learn about MSG? The text about 3Jcc ends with a vague statement.

We have now added an extra comment, which states that access to both the $^{13}C^{\delta1}$ chemical shift and the $^{13}C^{\delta1}$-$^{13}C^{\alpha}$ scalar coupling allows a full characterisation of the rotameric sampling about the χ_2 angle.

"Access to both the $^{13}C^{\delta 1}$ chemical shift and the $^{13}C^{\delta 1}$-$^{13}C^{\alpha}$ scalar coupling allows a full characterisation of the rotameric sampling about the χ_2 angle. For example, a large $^{13}C^{\delta 1}$ chemical shift and a small $^{13}C^{\delta 1}$-$^{13}C^{\alpha}$ coupling for I200 in MSG show that this residue is restrained in a rare gauche+ state (see Supplementary Table 4),[25] in agreement with the crystal structure of MSG."

T1 and T1rho experiments are hidden in the SI and not really discussed. Were they designed (and required) to get R1 and R2 values for CEST analysis? When reading the main text, the reader should at least learn that they exist.

The R_1 and $R_{1\text{rho}}$ (R_2) rates were measured to substantiate the discussions (*1*) That the small R_1 rates generally observed for ^{13}C necessitate a longer recycling delay and (*2*) the small R_2 rates leads to very sharp signals and allow for a large set of experiments to be carried out. We have now added extra sentences and commented more on the observed relaxation.

"The lack of directly bonded protons and absence of efficient relaxation pathways for aliphatic ^{13}C in highly deuterated proteins dramatically reduces the ^{13}C relaxation rates. For T4L L99A the isoleucine $^{13}C^{\delta 1}$ non-selective longitudinal relaxation rates, R_1, range between 0.12 s^{-1} and 0.24 s^{-1} at 278 K and a field of 14.1 T (see Supplementary Table 1 and Supplementary Figure 5."

" ... leads to small transverse relaxation rates, between 2.7 s^{-1} and 8.8 s^{-1} for isoleucine $^{13}C^{\delta 1}$ in T4L L99A at 278 K and 14.1 T (see Supplementary Table 2 and Supplementary Figure 7),"

(ii) The following must be resolved, clarified, or simplified to facilitate reproducibility.

For clarity, and to make sure that no parameter has been left out, I would duplicate some information from the Method section in the supporting information, as relevant, so that a complete description can be found in one place, in the SI. Notably, information for the "root" pulse sequence is spread between Figure 1, the Method, and SI Figure S2. You should consolidate all information needed for reproducibility in the caption of S2, including a (very brief) description of the IPAP block.

We have now included all the information for the core sequence needed for reproducibility in Supplementary Figure 2 and the legend as suggested by the reviewer. This includes a brief description of the IPAP block.

"The IPAP block is shown below the pulse sequence, where the two sub-spectra required for IPAP were acquired before the t_1 loop."

Rather than giving examples, please provide a table for all 13C and 2H carrier frequencies for all residue-specific moieties. I imagine some effort went into determining these numbers and the quality of the correlation maps will depend on them.

We have now added a table in supplementary information (Supplementary Table 5), where all carrier frequencies for reproducing the experiments have been summarised. The site-specific one-bond scalar coupling constants have also been listed.

Please specify that chemical shifts (and carrier frequencies) are referenced with respect to DSS (if they are). This information is particularly relevant for implementing the pulse sequence as some Bruker installations come with TMS when others come with DSS as default (irrespective of subsequent corrections during analysis).

All carrier frequencies are referenced with respect to DSS. This has also been commented on in Supplementary Table 5.

"a) All chemical shifts are referred to DSS (4,4-dimethyl-4-silapentane-1-sulfonic acid). For referencing to TMS (tetramethylsilane), which is often used by Bruker spectrometers, 2.65 ppm is subtracted from all ^{13}C chemical shifts listed. b) The ^{2}H carrier frequencies are obtained from the BMRB"

Please specify that the 13C carrier is shifted according to 90 shaped pulses on Ct and Cp in all cases unless otherwise indicated (if true).

This has now been specified in the legend to Supplementary Figure 2.

"The ^{13}C carrier is placed in the middle of the $^{13}C_t$ region, with the exception of the indirect chemical shift evolution period, where the carrier is placed in the middle of the $^{13}C_p$ region. As such, all frequency-selective pulses are applied on-resonance, except for the two 180° $^{13}C_p$ pulses in the IPAP block that are frequency-shifted by a linear phase modulation (see Table S5)."

Line 225: include "TCI" (I believe the probe is a TCI?)

The reviewer is correct, a TCI probe was used. This has now been included.

"… an HCN inverse TCI coldprobe equipped …"

When describing the encoding of indirect dimensions, the term "real points" would imply that as many imaginary points were collected. If so, please state explicitly. If not, replace with "complex points".

The number of points recorded was unfortunately given using standard NMR jargon, where the term 'real points' refers to real points plus imaginary points, this is the number also referred to as 'TD' on Bruker spectrometers. We have now changed throughout the manuscript to provide the number of complex points (half of the previous numbers, but the exact same data).

In S5, the authors measure recovery from transverse magnetization rather than inversion recovery. A little note and reference is needed here.

It is correct that we have measure the R_1 rates, by first purging the residual magnetisation and subsequently observing recovering back towards equilibrium. In this

way, the curves can be used directly to judge the length of the recycling delay needed. We have added the following to Supplementary Figure 5:

"Residual longitudinal magnetisation is initially purged by the 90º – g_1 element, such that recovery curves start at approximately zero intensity. These recovery curves can therefore directly be used to judge the recycling delay needed for other experiments, such as Supplementary Figure 2."

In all relaxation experiments, provide the exact values of relaxation times (mixing times) and the order in which they were acquired (including if interleaved mixing time/quadrature acquisition was used).

These have now been added.

In Supplementary Table 1:

" ... 11 relaxation delays {0.5 s, 7.0 s, 1.0 s, 6.0 s, 1.5 s, 5.0 s, 2.0 s, 4.0 s, 2.5 s, 3.5 s, 3.0 s} (14.1 T) or 10 relaxation delays {0.001 s, 8.0 s, 0.5 s, 6.0 s, 1.0 s, 4.0 s, 2.0 s, 3.0 s, 4.0 s, 1.0 s} (18.8 T)."

In Supplementary Table 2:

" ... 21 relaxation delays:{0.001 s, 0.104 s, 0.002 s, 0.096 s, 0.004 s, 0.088 s, 0.008 s, 0.080 s, 0.012 s, 0.072 s, 0.016 s, 0.064 s, 0.020 s, 0.056 s, 0.024 s, 0.048 s, 0.028 s, 0.040 s, 0.032 s, 0.008 s, 0.032 s, 0.096 s}. "

To show the order in which spectra were recorded (interleaved), the following was added to the legend of Supplementary Figure 5 and 7:

" ... Spectra were recorded in an interleaved manner with the two IPAP sub-spectra recorded as the inner loop, τ_{relax} as the middle loop, and the t_1 array as the outer and last loop."

Typos:
Line 255: giving -> given.

SI: Figure S1: swap Cd-Ce with Ce-Cd (for consistency with your notation elsewhere).

S9: "during rest of" -> "during the rest of"

These typos have all been corrected.

Reviewer 3:

a) As discussed in the paper, the pulse sequence starts with magnetization from 13C and detects magnetization on 13C. This makes the pulse sequence very insensitive. However, they claimed to measure experiment within 12 mins by applying spectral folding/aliasing. I think they should report this along with the sample concentrations to prevent any confusion.

We completely agree with this reviewer and with reviewer 1, that the concentration of the T4L L99A sample should be included. We have now explicitly included the concentration of the T4L L99A samples used (also see above).

In Figure 2:
" ... obtained on a 1.4 mM sample"

In the methods section:
"The NMR sample contained 1.4 mM protein in 95%/5% $^{1}H_2O$ / $^{2}H_2O$ for the uniformly [$^{2}H^{13}C^{15}N$] labelled sample and 1.5 mM protein in 100% $^{2}H_2O$ for the [$^{1}H^{13}C$-Lys,Arg; $^{2}H^{12}C$] labelled sample."

In the legend to Supplementary Figure 3 and 4:
" ... using a 1.4 mM sample of T4L L99A .. "

b) They should also discuss the effect of homonuclear coupling in the measurement of T1p and CEST. There is a paper by Kay et.al which talks about in general negligible effect of 13C-13C homonuclear coupling in CEST measurements. Are there any studies for T1p?

We had previously mentioned in the method section that ^{13}C-^{13}C homonuclear couplings were taken into account in the analysis of the CEST data: " …calculated intensities were obtained by evolving the spin system according the Liouvillian described previously[5,17,33,34], taking into account the one-bond ^{13}C-^{13}C scalar coupling...". We have now added additional information in the method section:

" ... one-bond ^{13}C-^{13}C scalar coupling, which generally leads to a simple line-broadening of the CEST profiles."

For the measurements of $T_{1\rho}$ relaxation times, it is correctly pointed out by the reviewer that the ^{13}C-^{13}C homonuclear couplings could potentially interfere with the measurements. We were inspired by the paper by Yamazaki *et al.* (1994), J. Am. Chem. Soc. 116 p 8266-8278, where alpha-^{13}C transverse relaxation rates were measured in uniformly ^{13}C labelled proteins using a $T_{1\rho}$ type experiment. It was shown by Yamazaki *et al.* that Hartmann-Hahn transfers are not interfering with the obtained relaxation rates for the parameters used.

Our own simulations (using a full 16 dimensional spin-basis; Hard et al, DOI: 10.1006/jmre.1997.1252) show that systematic errors in the obtained $T_{1\rho}$ rates due to Hartmann-Hahn transfers and homonuclear couplings are less than 2% over the range of parameters used here.

We have now included the reference the paper by Yamazaki *et al.* in Supplementary Figure 7, where similar simulations were shown (for alpha-^{13}C – beta-^{13}C) couplings. We do not think that an in-depth explanation of our simulations, which are in agreement with Yamazaki *et al.*, will benefit the readership of Nature Communications.

c) The decoupling sought by IPAP depends on the 13C-13C scalar coupling. Is it safe to assume 35 Hz for all side chains 13C-13C coupling? If this is not the case it can distort the line shapes.

We have now measured the $^1J_{CC}$ couplings for the different residue types and given these in Supplementary Table 5. Firstly, the standard derivation of the derived $^1J_{CC}$ within each experiment is small (<1.1 Hz), and no distortion to the line shape is therefore expected. As such, it is safe to assume 35.5 Hz for all residues although slightly better signal-to-noise is obtained by using the site-specific values.

d) The recommended interscan delay is 3T1 to 5T1 which amount to 30 to 50 seconds in these experiments. However, they have recorded with an interscan delay of 4 and 10 seconds. This would have impacted the sensitivity.

It is correct that it is recommended to generally use interscan delays of ~3 T_1 in order to obtain NMR signal intensities that are independent of site-specific T_1. The maximum sensitivity per unit time is, however, observed for interscan delay of d_1 = 1.26 T_1. It is thus correct that slightly better sensitivity per unit time could be obtained by increasing the interscan delay from 4 seconds to ~ 5-10 seconds (depending on site) for the T4 L99A sample. One does, however, need to keep in mind that increasing the interscan delay will increase the total length of the experiment, if the same resolution is to be obtained.

The experiments were run with the full phase cycle (4 steps for the 2D spectra) and with the maximum resolution possible in the t_1 dimension within the constant time. We have now added a comment about these considerations in the supplementary material.

In Supplementary Figure 2:
"Optimal sensitivity per unit time is achieved with d_1 ~ 1.26/R_1."

In Supplementary Figure 5:

" ... and thus for the T4L L99A protein studied here, an interscan delay between 5 s and 10 s results in the maximum sensitivity per unit time."

… They may improve sensitivity per unit time by flipping magnetization for less than 90 degrees.

We completely agree with the reviewer that optimising the sequence for the flip angle in line with the original idea by R.R. Ernst, BEST or SoFAST would be desirable. However, due to the significant number of selective pulses and IPAP filter we have unfortunately not yet been able to implement this idea properly.

e) Finally, authors can write a few lines on what extra information such correlations

can provide which cannot be got from by existing experiments which study 1H, 13C correlation of terminal methyls. Of course, one thing is that the indirect dimension has better chemical shift dispersion by using 13C instead of 1H.

Each experiment provide direct access to the chemical shift of two aliphatic ^{13}C. We have previously shown that the aliphatic side-chain ^{13}C chemical shifts report on the side-chain structure and conformational sample. Although these are methods that we are currently working on and improving, we have added the following sentence:

" ... Moreover, as compared to ^{1}H-^{13}C spectra, the ^{13}C-^{13}C correlation maps directly provide the chemical shift of two aliphatic ^{13}C that are both known to report on the structure and sampling of side chains"

We have added the following sentence about the better resolution:

" Another striking advantage of ^{13}C-^{13}C correlation spectra compared to ^{1}H-^{13}C spectra is the substantially better chemical shift dispersion in the directly detected dimension resulting in significantly better peak separation, (see Supplementary Figure 6 for a comparison)."

Minor Comments:

1. On page 4 "..characterize many side chains." The number is 6, and 5 without interference from other amino acids.

We have now in the abstract specified that six different amino acid types can be characterised:

" .. the side chains of six different amino acid types ..."

We also changed the word "many" to *"six"* in the sentence that the reviewer is referring to.

2. Fig S5 for measuring T1, the first pulse is 90 degrees. In the inversion recovery, the magnetization is flipped to 180 degrees.

We did not attempt to obtain an 'inversion recovery experiment', which is why we named the figure "Measurements of longitudinal R_1 rates". Although R_1 rates are often measured using the inversion recovery experiment, we chose here to use the setup in Supplementary Figure 5 to also directly allow for an assessment of the interscan delay required for the experiments. We have added the following in Supplementary Figure 5:

"Residual longitudinal magnetisation is initially purged by the 90° – g1 element, such that recovery curves start at approximately zero intensity. These recovery curves can therefore directly be used to judge the recycling delay needed for other experiments, such as Supplementary Figure 2."

3. Fig s4 (b), two peaks are assigned 145. One of them should be 148.

This has now been corrected.

4. Please mention the units in Table S3.

This has now been corrected

5. On page 11, "..experimental details are giving in the legend" should be "..are given"

This has now been corrected.

6. The caption of Fig S12, "fellows the original idea" should be "follows"

This has now been corrected

7. The work of Dominique Marion on out and back TOCSY should be mentioned. J Biomol NMR. 2015 Dec;63(4):389-402

This reference has now been added:

“TOtal Correlated SpectroscopY (TOCSY) NMR experiments are typically used to aid side-chain chemical shift assignment (- ref 28 -) of proteins and …”

REVIEWERS' COMMENTS:

Reviewer #1 (Remarks to the Author):

The authors have fully addressed all my prior concerns and I now enthusiastically recommend this work for publication in Nature Commmunications.
